# Characterization of an isogenic *bimA* mutant in the ATS2021 strain of *Burkholderia pseudomallei*

**DOI:** 10.1128/iai.00727-25

**Published:** 2026-06-18

**Authors:** Christopher K. Cote, Sherry Mou, Michael L. Davies, Kevin D. Mlynek, Christopher P. Klimko, Sergei S. Biryukov, Nathaniel O. Rill, Jennifer L. Dankmeyer, Taloria K. Wheeler, Carlos I. Rodriguez, Brian A. Smith, Melissa Hunter, Elsie I. Martinez, Christina E. Douglas, Cheryl Taylor-Howell, Taalin S. Hoj, Christopher P. Stefan, Ju Qiu, Xiankun Zeng, Joel A. Bozue, Nancy A. Twenhafel, Charles J. Shoemaker, David DeShazer

**Affiliations:** 1 Bacteriology Division, US Army Medical Research Institute of Infectious Diseases (USAMRIID) 19919 , Frederick, Maryland, USA; 2 Diagnostic Systems Division, US Army Medical Research Institute of Infectious Diseases (USAMRIID) 19919 , Frederick, Maryland, USA; 3 Biostatistics Branch, US Army Medical Research Institute of Infectious Diseases (USAMRIID) 19919 , Frederick, Maryland, USA; 4 Pathology Division, US Army Medical Research Institute of Infectious Diseases (USAMRIID) 19919 , Frederick, Maryland, USA; Stanford University School of Medicine, Stanford, California, USA

**Keywords:** *Burkholderia *intracellular motility A protein, BimA, *Burkholderia pseudomallei*, neurological infection, mice, aerosol

## Abstract

Encephalomyelitis is uncommon but is associated with *Burkholderia pseudomallei* strains whose *bimA* gene, encoding an actin-based motility protein, resembles *bimA* from *Burkholderia mallei* (designated *bimA_Bm_*). We previously characterized the virulence of *B. pseudomallei* wild-type ATS2021 (*bimA_Bm_*). In a mouse model of ATS2021 aerosol exposure, lesions and inflammatory cytokines in the brain were seen early post-exposure and reached the central nervous system (CNS) through the olfactory nerve. In the current study, the *bimA* allele in the wild-type ATS2021 was replaced with the *bimA_Bp_*, and virulence was assessed by estimations of LD_50_, histopathological analyses, and bacterial dissemination. ATS2021 *bimA_Bp_* reached the CNS but was significantly attenuated, with higher LD_50_ estimates, altered bacterial dissemination, and reduced neuroinflammation. The immune response in lungs, spleens, and brains from mice exposed to aerosols indicated either strain induced shared and divergent immune patterns. Finally, the evaluation of the host response in brains via NanoString transcriptomic platform analyses suggested that the wild-type ATS2021 induced substantial changes in astrocyte and microglial activation, oligodendrocyte function, and overall inflammatory signaling, compared to the mutant *bimA_Bp_* bacteria. These results support a direct role of the *bimA_Bm_* gene product for bacterial dissemination after inhalation.

## INTRODUCTION

*Burkholderia pseudomallei* is the etiologic agent of melioidosis, a disease that occurs in many tropical and subtropical countries around the world (1, 2). It is highly endemic in northern Australia and Southeast Asia but is an emerging disease in South Asia, Africa, and the Americas. *B. pseudomallei* infection is an emerging threat in North America because of the declaration of endemicity in the Gulf Coast region of the United States and infections associated with imported animals or products (3–5). The organism is a gram-negative bacillus and an environmental saprophyte that causes infections in humans and animals via percutaneous inoculation, ingestion, or inhalation of contaminated soil or water. Human melioidosis is protean in nature as many organs can be infected, and the clinical presentation can vary widely to include skin and soft tissue infection, spleen and liver abscess, genitourinary infection, prostatic abscess, bone and joint infection, pneumonia, encephalomyelitis, meningitis, brain abscess, and septicemia (6, 7). The mortality rate ranges from approximately 10%–40% depending on the availability of adequate medical care in distinct geographical locations, route of infection, infectious dose, and virulence of the infecting strain. While apparently healthy individuals can be infected, many patients have an underlying risk factor such as diabetes mellitus, chronic lung disease, chronic liver disease, excessive alcohol consumption, and thalassemia (8). There are currently no licensed vaccines for melioidosis, and the two-phase antimicrobial therapy used to treat patients lasts at least 3–6 months (9, 10).

*B. pseudomallei* is a facultative intracellular pathogen that can escape epithelial and phagocytic cell endocytic vesicles, replicate within the cytosol, and mediate intracellular and intercellular movement by polymerizing actin monomers (G-actin) into actin filaments (F-actin) (11, 12). The *Burkholderia* intracellular motility A (BimA) protein is a trimeric, autotransported protein localized at one pole of the bacterium and essential for generating the iconic actin “comet tails” that produce membrane protrusions promoting fusion with adjacent cells and multinucleated giant cell (MNGC) formation (13–16). Thus, BimA provides *B. pseudomallei* a mechanism to evade immune surveillance while maintaining an intracellular niche that contains sufficient nutrients for survival and persistence within the host. The glanders pathogen, *Burkholderia mallei*, also harbors a *bimA* gene that can facilitate intracellular and intercellular motility (16). *B. mallei* is a host-adapted clone of *B. pseudomallei*, and the genes that they share are approximately 99% identical. Interestingly, the genomic sequences of *B. mallei* ATCC 23344 and *B. pseudomallei* K96243 revealed that the 5′ ends of the *bimA* gene in *B. mallei* (*bimA_Bm_*) and *B. pseudomallei* (*bimA_Bp_*) are unique (17). Despite the unique domain structure of the N-termini of the BimA_Bm_ and BimA_Bp_ proteins, both can elongate and bundle actin filaments in a mechanism that mimics Ena/VASP actin polymerases found in eukaryotes (14, 15).

Neurological melioidosis, first described in humans in 1977, is a rare manifestation of disease (1.5%–5% of cases) that has a mortality rate of approximately 20% (18). A relatively large percentage of survivors display some degree of lifelong neurological impairment. During the past decade, multiple studies have found that patients infected with *B. pseudomallei* strains harboring the *bimA_Bm_* allele are more likely to present with neurological melioidosis and more commonly die or have long-term disability compared to those infected with *B. pseudomallei* containing the *bimA_Bp_* allele (19–24). *B. pseudomallei bimA_Bm_* isolates were also significantly associated with sepsis and mortality in Sri Lankan patients (25). The *bimA_Bm_* allele is present in isolates from Sri Lanka (18.5%), Australia (~12%), and India (4.5%) but has not yet been found in isolates from Thailand (21, 24, 26). Interestingly, *B. pseudomallei bimA_Bm_* strains were more virulent than *B. pseudomallei bimA_Bp_* strains in both BALB/c and C57BL/6 mice (22, 27). It is important to note, however, that *B. pseudomallei* has an open genome, and each isolate is predicted to have >100 novel genes (28). Thus, it is difficult to assess whether the virulence differences are due exclusively to the presence of the *bimA_Bm_* allele or the association of the *bimA_Bm_* allele with other unique virulence genes potentially present.

The objective of this study was to construct and evaluate the pathogenesis and virulence of an isogenic strain of *B. pseudomallei* harboring a *bimA_Bm_* or a *bimA_Bp_* allele. The *bimA_Bm_* strain utilized for these experiments was *B. pseudomallei* wild-type ATS2021, the source of a 2021 melioidosis outbreak in the United States involving patients from four states that used a contaminated aromatherapy spray (29). The spray was imported from India, and the isolate caused neurologic melioidosis and resulted in two deaths. *B. pseudomallei* ATS2021 was also found to be highly virulent in the inhalational BALB/c and C57BL/6 mouse models of melioidosis (3).

In the current study, the original ATS2021 *bimA_Bm_* allele was replaced with the *bimA_Bp_* allele from the prototypic strain, K96243, creating the recombinant ATS2021 *bimA_Bp_* strain. The two strains were evaluated for actin-based motility and MNGC formation in RAW264.7 cells, plaque formation in A549 cells, replication and survival in RAW264.7 and C8-D1A (an astrocyte-like cell line) cells, and *in vitro* formation of bacterial biofilms. ATS2021 *bimA_Bp_* was also assessed for virulence in C57BL/6 mice, bacterial burden and dissemination, cytokine levels in tissues, histopathological analysis, and impact on host neurological gene expression. When compared with the previously published data for the wild-type ATS2021 strain (3), the results definitively confirm that the BimA_Bm_ protein is a *B. pseudomallei* virulence factor that confers an appreciable neurotropic disease manifestation.

## MATERIALS AND METHODS

### Construction of the *bimA_Bp_* mutant strain

The oligonucleotide primers *bimA*-fwd (5′-GCTAGCAAGTGCAGCGATTTCTGACG-3′) and *bimA*-rev (5′-GCTAGCGTTGAACTCGTCCATGTAGG-3′) were used with genomic DNA from *B. pseudomallei* K96243 to generate a PCR product of 2,304 bp. The PCR product was cloned into pCR2.1-TOPO, generating the plasmid pCR2.1-*bimA_Bp_*. The *Nhe*I fragment was removed and cloned into the gene replacement vector pMo130, digested with *Xba*I and *Nhe*I. The resulting plasmid, pMo130-*bimA_Bp_*, was electroporated into *Escherichia coli* S17-1 and conjugated with *B. pseudomallei* ATS2021 for 8 h. Kanamycin (1 mg/mL) was used to select for merodiploid formation, and polymyxin B (25 mg/mL) was used to counter-select *E. coli* S17-1. *B. pseudomallei* ATS2021 derivatives harboring the *bimA_Bp_* allele rather than the wild-type *bimA_Bm_* allele were identified by colony PCR.

### Eukaryotic cell lines

Murine macrophage-like cells (RAW 264.7 [ATCC, TIB-71]), murine astrocyte cells (C8-D1A [ATCC, CRL-2541]), and human lung epithelial cells (A549 [ATCC, CCL-185]) were used in this study. Unless indicated otherwise, the cells were cultured and maintained in cell culture medium supplemented with 10% heat-inactivated fetal bovine serum (Hyclone, Logan, UT, USA), 2 mM GlutaMAX (Gibco, BRL, Grand Island, NY, USA), and 1% non-essential amino acids (Sigma Chemical Co., St. Louis, MO, USA). Dulbecco’s modified Eagle medium (Corning, Mediatech, Inc., Manassas, VA, USA) was used for the RAW 264.7 and C8-D1A astrocyte cell lines, while Eagle minimum essential medium (MEM, Corning, Mediatech Inc., Manassas, VA, USA) was used for the A549 cell line. Throughout the study, the cells were incubated at 37°C in a humidified incubator in the presence of 5% CO_2_.

### Intracellular replication assay

The net intracellular replication of *B. pseudomallei* in macrophage-like cells and astrocyte cells was assessed as described previously (30), with some modifications. RAW 264.7 cells were seeded at 2 × 10^5^ cells/well, and C8-D1A cells were seeded at 8 × 10^4^ cells/well in 24-well tissue culture plates. Both cell lines were infected ~48 h later with wild-type *B. pseudomallei* ATS2021 or ATS201 *bimA_Bp_* at a multiplicity of infection (MOI) of 10. After a 2-h infection at 37°C, the cells were washed with PBS, and medium containing kanamycin (1 mg/mL) was added to kill extracellular bacteria. The infected cells were subsequently lysed at 2, 4, 6, 8, and 10 h post-infection with PBS containing 0.1% Triton X-100. Viable intracellular bacteria were quantitated by spreading serial dilutions onto LB broth Lennox agar plates. Colony-forming units (CFUs) were assessed after 48-h incubation at 37°C. The CFU counts were compared between the bacterial strains using a negative binomial generalized linear model.

### Fluorescence staining and confocal microscopy of *B. pseudomallei* and F-actin in infected RAW 264.7 cells

RAW 264.7 cells were cultured on 12 mm diameter glass coverslips (1.5, Electron Microscopy Sciences, Hatfield, PA, USA) placed in 12-well plates. After 36 h, the cells were infected with *B. pseudomallei* at an MOI of approximately 10. After 2 h of incubation, the extracellular bacteria were removed by washing the cells with PBS, and fresh culture medium containing kanamycin (1 mg/mL) was added. At the experimental endpoint, the infected cells were washed with PBS and then fixed with 10% formalin (Fisher Scientific) for 72 h. The fixed cells were washed with PBS before permeabilization with 0.5% (vol/vol) Triton X-100 in PBS for 1 h. To minimize nonspecific binding, the cells were blocked for 2 h with 7% normal goat serum, 0.2% (wt/vol) bovine serum albumin (Sigma Chemical Co.), and 0.5% Triton X-100 in PBS at room temperature. Subsequently, bacteria were stained with rabbit polyclonal anti-*B*. *pseudomallei* antibody (USAMRIID) at a dilution of 1:1,000 overnight at 4°C, followed by Alexa-Fluor 488-conjugated anti-rabbit immunoglobulin (Life Technologies, Carlsbad, CA, USA) at a dilution of 1:700. Actin filaments and DNA were stained using Alexa Fluor 568-conjugated Phalloidin (Life Technologies) at a dilution of 1:700 and 4′,6-diamidino-2-phenylindole (Life Technologies) at a dilution of 1:400, respectively. Images were captured on an LSM 880 Confocal Microscopy (Zeiss, Oberkochen, Germany) and analyzed using ImageJ software (National Institutes of Health, Bethesda, MD, USA).

### Plaque formation assay

*B. pseudomallei*-induced plaque assays were performed as described earlier (31) with some modifications. A549 cells were seeded at 1 × 10^6^ cells/well in 6-well cell culture plates. After 36 h, cells were infected with *B. pseudomallei* at an MOI of 1 and incubated at 37°C with 5% CO_2_ for 2 h. After 2 h of incubation, the infected cells were washed and replaced with medium containing kanamycin (1 mg/mL). The plates were incubated at 37°C with 5% CO_2_ for at least 22 h. At the experimental endpoint, the infected cells were washed with PBS and fixed with 10% formalin for 72 h. The fixed cells were then washed with PBS prior to staining. Plaques were stained with 1% (wt/vol) crystal violet in 20% (vol/vol) methanol and enumerated. Plaque-forming efficiency was calculated as the number of plaques divided by the bacterial CFU added per well.

### Growth analysis

Strains were grown in Luria broth (Difco, Thermo Scientific, Rockville, MD, USA) + 4% glycerol (Sigma, St. Louis, MO, USA) (LBG) or in glycerol-tryptone broth (GTB) containing 4% glycerol, 1% tryptone (Bacto Gibco, Thermo Scientific, Rockville, MD, USA), and 0.5% NaCl (Sigma, St. Louis, MO, USA), with shaking at 37°C for 18 h. Following incubation, cultures were normalized to an OD_600_ of 0.2 in PBS (Corning, Sullivan Park, NY, USA) and diluted 1:10 into LBG in a CoStar polystyrene 96-well plate. Growth was then assayed by OD_600_ reading every 30 min using a Tecan Spark (Tecan Systems, Morgan Hill, CA, USA) microplate reader at 37°C with orbital shaking. Absorbance values were determined using the average of at least four technical replicate wells and subtracting the medium-only background as determined by the sterility control. All data reported are the result of at least three biologically independent experiments.

### Biofilm analysis

Determination of biofilm production from *B. pseudomallei* strains was performed as previously described (3). Briefly, overnight cultures grown in LBG were normalized to an OD_600_ of 0.2 and seeded into 96-well plates containing fresh LBG using a 1:10 dilution. Sterility wells were included in each experiment, and peripheral wells were avoided to minimize edge effect. Plates were then incubated statically at 37°C for 24 h. To detect biofilm, planktonic cells were aspirated, and wells were washed three times with PBS, followed by fixation with 100% ethanol for 30 min at room temperature. A solution of 0.1% crystal violet (wt/vol) was added to each well for 15 min and washed three times with PBS to remove residual stain. The crystal violet stain was solubilized in 33% acetic acid, and the OD_600_ was measured to quantify crystal violet staining as an indicator of biofilm formation. When necessary, samples were diluted 1:10 in 33% acetic acid to ensure that OD readings were within the linear range. At least four technical replicates were averaged in each experiment. All data reported are the result of at least three individual experiments.

### Animals and challenge conditions

Female C57BL/6 mice were obtained from Charles River (Frederick, MD, USA) and were 7–10 weeks of age at the time of exposure to aerosolized *B. pseudomallei*. Groups of mice were challenged with *B. pseudomallei* ATS2021 *bimA_Bp_* by a whole-body aerosol exposure system. The bacterial cultures were started from a freezer stock and grown for approximately 16 h in GTB at 37°C at 150 RPM. The optical density was determined at OD_620_, the culture was centrifuged for 10 min at 5,000 RPM and then resuspended in fresh GTB. The OD_620_ was determined again, and the culture was diluted to the desired concentration to perform the aerosol procedure. For the exposures, mice were transferred to wire mesh cages and placed in a whole-body aerosol chamber within a class three biological safety cabinet located inside a BSL-3 laboratory. Mice were then exposed to aerosols of *B. pseudomallei* suspension created by a three-jet collision nebulizer (32). Samples were collected from the all-glass impinger vessel and analyzed bacteriologically to determine the inhaled dose of *B. pseudomallei* in CFU.

### Clinical observations and sample collections

Challenged mice were observed at least once daily for 60 days for clinical signs of illness, as described previously (32). Early intervention endpoints were used during all studies, and mice were euthanized when moribund, according to early endpoint criteria. Animals were scored on a scale of 0–9: 0–2, no significant clinical signs (e.g., slightly ruffled fur); 3–4, significant clinical symptoms such as subdued behavior, hunched appearance, absence of grooming, hind limb issues of varying severity and/or pyogranulomatous swelling of varying severity (increased monitoring was warranted); and >5, distress. Those animals receiving a score of >5 were euthanized with a pentobarbital-based euthanasia solution given intraperitoneally. Animals that survived were euthanized at the study endpoint and necropsied for tissue collection for bacteriological analyses.

### Bacteriology

The number of viable bacteria present in tissues of mice was determined on selected time points post-challenge and for survivors at the study endpoint. The samples collected from necropsied mice included blood, lungs, spleen, and brain. They were weighed and homogenized with disposable PRECISION homogenizers (Covidien, Dublin, Ireland); the CFU of the homogenates was determined on sheep blood agar plates (Remel, Thermo Scientific, Rockville, MD, USA). Undiluted homogenate and 10-fold serial dilutions in PBS were plated in duplicate to determine sterility. The values reported were the geometric mean and geometric standard deviation of CFU/mL of blood and CFU/g of organ. The limit of detection was approximately 50 CFU/mL blood or 5 CFU/organ. Due to the small amount of blood analyzed, blood CFU data are presented as the percentage of mice that were bacteremic based on the presence of bacterial growth on a single plate.

### Histopathological and immunohistochemistry analyses

Pathological assessment was performed on a subset of mice from each group of mice exposed to approximately 204, 1,563, or 15,566 CFU of aerosolized *B. pseudomallei* ATS2021 *bimA_Bp_*. Necropsies were performed, and tissues were processed following 21 days in 10% buffered formalin. The tissues were trimmed, processed, embedded in paraffin, cut by microtomy, stained, coverslipped, and screened. Tissue blocks and slides were produced and stained with hematoxylin and eosin. Immunohistochemistry (IHC) was performed on select animals using the BOND RX Automated Stainer (Leica Biosystems, Lincolnshire, IL, USA). A rabbit polyclonal anti-*Burkholderia* antibody (antibody #351; USAMRIID) was used at a dilution of 1:5,000. The sections were stained with hematoxylin (nuclei), followed by counterstaining with eosin (extracellular matrix and cytoplasm), and coverslipped. Severity scores indicate what percentage of the tissue examined was affected and subjectively determined by the study pathologist as follows: 0, none; 1, minimal 0%–10%; 2, mild 10%–25%; 3, moderate 25%–50%; 4, marked 50%–75%; and 5, severe 75%–100%. Similarly, the amount of IHC positivity was subjectively determined by the study pathologist and scored as follows: 0, negative; 1, minimal 0%–10%; 2, mild 10%–25%; 3, moderate 25%–50%; 4, marked 50%–75%; or 5, severe 75%–100%. Animal total scores were achieved by adding each severity score. Total animal group scores were achieved by adding all animal total scores.

IHC for IbaI, a marker for microglia and macrophages, was performed using the Dako Envision system (Dako Agilent Pathology Solutions, Glostrup, Denmark). Briefly, after deparaffinization, peroxidase blocking, and antigen retrieval, sections were covered with a rabbit anti-Iba1 antibody (ab178846, Abcam, Waltham, MA, USA) at a dilution of 1:500 and incubated at room temperature for 45 min. They were rinsed, and the peroxidase-labeled polymer (secondary antibody) was applied for 30 min. Slides were further rinsed and a brown chromogenic substrate 3,3′ diaminobenzidine solution (Dako Agilent Pathology Solutions) was applied for 8 min. The substrate-chromogen solution was rinsed off the slides, and the slides were counterstained with hematoxylin and rinsed. The sections were dehydrated, cleared with Xyless, and then coverslipped. The samples were then scored as Activated (A, some or many [25%–100%] microglia appear activated as determined by bushy and/or amoeboid phenotype), Partially Activated (PA, few to some [1%–25%] of microglia appear activated), or Ramified (R, most or all [75%–100%] microglia display a ramified and/or hyper-ramified phenotype).

### Cytokine analyses

Cytokines were quantified in tissue homogenates across select days 1–10 post-challenge in mice with low-challenge dose (107 CFU wild-type ATS2021 or 204 CFU mutant *bimA_Bp_* ATS2021); days 1–5 in mice with mid-challenge dose (1,149 CFU wild-type ATS2021 or 1,563 CFU mutant *bimA_Bp_* ATS2021); and days 1–3 in mice with high-challenge dose (4,488 CFU wild-type ATS2021 or 15,566 CFU mutant *bimA_Bp_* ATS2021). The challenge with wild-type ATS2021 was previously described (3). Of the 36 cytokines in the multiplex kit, quality control criteria were used to eliminate four from the analysis of brain and lung tissue and two for brain tissue only, leaving 32 cytokines for lungs and 30 for brains. At each time point, for lung and brain tissue, the cytokine levels after challenge with both strains were compared to each other and to the baseline levels measured in naïve mice. Geometric means and standard deviations were graphed for each group (*n* = 4).

### RNA extraction and NanoString data collection

Brain homogenates were inactivated with a 3:1 ratio of TRIzol LS (Thermo Fisher Scientific, Rockville, MD, USA), and total RNA was extracted as previously reported (33). Host gene expression data were collected by using the NanoString nCounter mouse Neuroinflammation panel on the SPRINT Profiler platform (Bruker Inc., Billerica, MA, USA) in an identical fashion to a previous report (3). In brief, 70 μL of hybridization buffer was added to the reporter code set to make a master mixture. Next, 8 μL of the master mixture was added to 50 ng of extracted host RNA and 2 μL of the capture code set. The resulting reaction mixture was incubated at 65°C for 17 h and then held at 4°C until the samples were placed on a NanoString SPRINT Profiler, where total fluorescent counts corresponding to target binding were collected. Count data were extracted from NanoString RCC files and analyzed by using the ROSALIND Bioinformatics suite (https://rosalind.bio). Normalized counts were generated by using the criteria provided by NanoString. ROSALIND follows the nCounter Advanced Analysis protocol (NanoString) of dividing counts within a lane by the geometric mean of the normalizer probes from the same lane. Housekeeping probes for normalization were selected based on the geNorm algorithm as implemented in the NormqPCR R library (34). Unless otherwise noted, significance of differentially expressed genes (DEGs) was based on greater than ±1.5-fold linear threshold expression and a *P* ≤ 0.05 relative to brain homogenates from the unchallenged mice group. Directed enrichment scores were generated by utilizing the gene set analysis module in ROSALIND, wherein changes in regulation within each defined gene set (based on differential change from naïve baseline) are scaled to a *t*-statistic distribution and summarized into a composite score that reflects changes in both relative magnitude and directionality (i.e., upregulated vs downregulated) for particular gene sets (i.e., NanoString annotated pathways).

### Statistics

The LD_50_ was estimated and compared under a Probit model with log transformation of the dose variable. Median survival time (time to death from disease or euthanasia in accordance with early-endpoint euthanasia criteria) and accompanying confidence limits, as well as mean survival time and standard error, were estimated by Kaplan-Meier survival methods and analyzed by the log-rank test. The survival rates at selected time points were compared by Fisher’s exact test. For biofilm, CFU, and Luminex data, pairwise treatment groups were compared by a linear mixed effects model. No multiplicity adjustment was applied. Analysis was implemented in SAS version 9.4 (Cary, NC, USA).

## RESULTS

### ATS2021 harboring a *bimA_Bp_* allele polymerizes host actin, generates membrane protrusions in RAW 264.7 cells, and forms plaques in A549 cells but shows differences in intracellular replication

**Fig 1:**
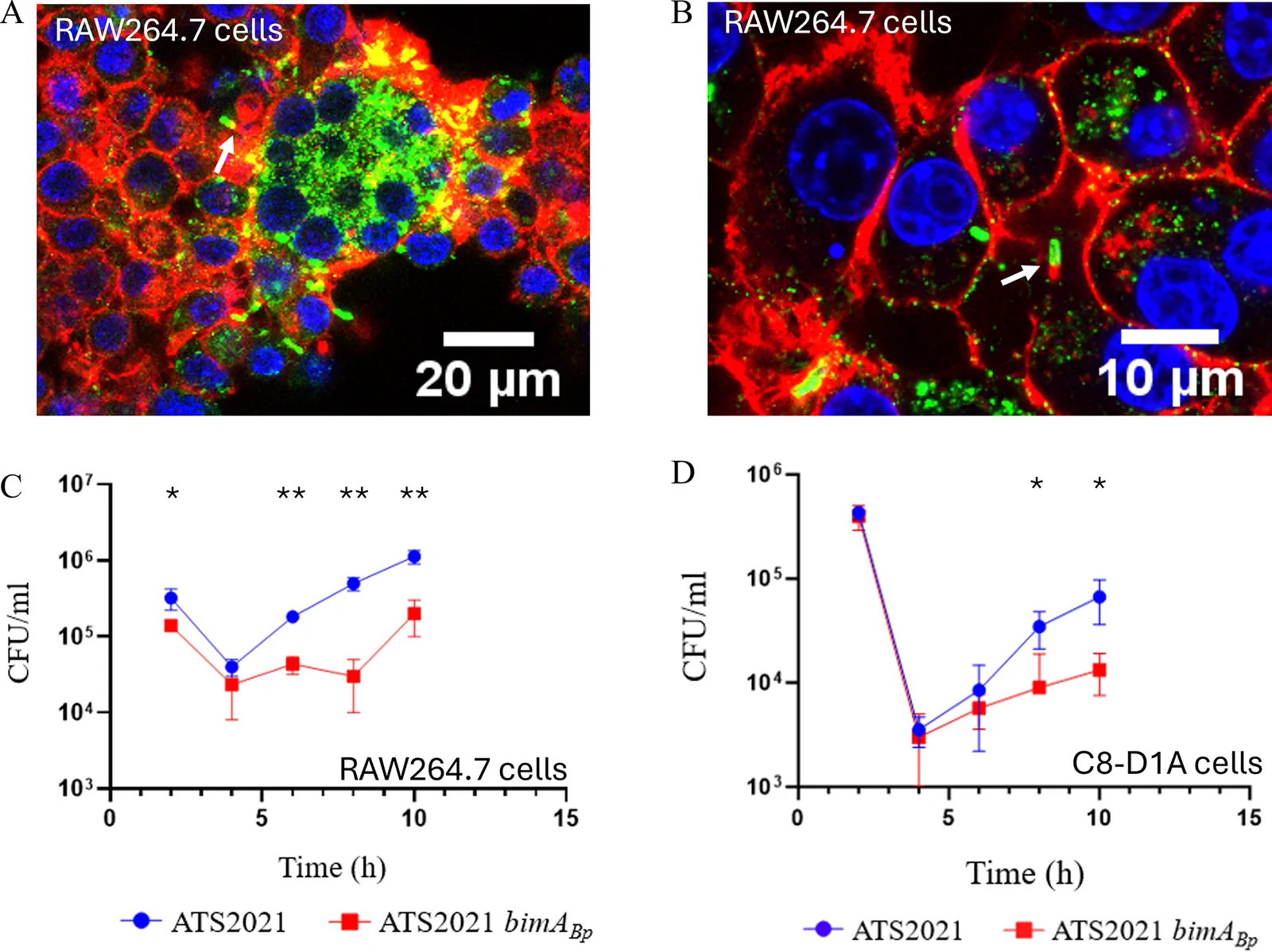
Assessment of intracellular survival and replication of the *bimA_Bp_* mutant. RAW 264.7 cells and C8-D1A cells were infected with *B. pseudomallei* for confocal microscopy imaging and assessment of the intracellular fate of the organism. RAW 264.7 cells were infected with *B. pseudomallei* ATS2021 (**A**) and ATS2021 *bimA_Bp_* (**B**) at an MOI of 10 and visualized at 10 h post-infection using immunofluorescence staining and confocal microscopy. F-actin is stained red, nuclei are stained blue, bacteria are stained green, and actin tails emanating from bacterial poles are indicated by white arrows. Both bacterial strains were used to infect RAW 264.7 cells (**C**) and C8-D1A cells (**D**) at an MOI of 10 and evaluated for intracellular growth and survival over a period of 10 h. The plots show the mean with SD bar. The CFU counts were compared using a negative binomial generalized linear model. *, *P* < 0.01; **, *P* < 0.001.

To compare the biological activity of BimA_Bm_ and BimA_Bp_ in an isogenic *B. pseudomallei* background, the native *bimA_Bm_* gene present in wild-type ATS2021 was replaced with the *bimA_Bp_* allele from the prototypic Thailand strain K96243 (35). As an intracellular pathogen, *B. pseudomallei* escapes endocytic vacuoles and moves within the host cytosol via BimA-mediated assembly of actin filaments, which leads to membrane protrusions that facilitate cell-to-cell spread and MNGC formation (12, 16). Murine macrophage-like RAW 264.7 cells were infected with wild-type ATS2021 or ATS2021 *bimA_Bp_*, and the cells were fixed at defined time points, stained using immunofluorescence, and imaged using confocal microscopy (Fig. 1). Both strains formed actin tails and membrane protrusions in RAW 264.7 cells (Fig. 1A and B), indicating that both BimA proteins function as predicted. MNGC formation was also observed in RAW 264.7 cells infected with either ATS2021 or ATS2021 *bimA_Bp_* (data not shown). Although ATS2021 *bimA_Bp_* did not affect actin filament assembly in RAW 264.7 cells, ATS2021 exhibited greater replication and survival than ATS2021 *bimA_Bp_* at the 6, 8, and 10 h post-infection time points (Fig. 1C). Since strains harboring the *bimA_Bm_* allele are often associated with neurological melioidosis, C8-D1A murine astrocyte cells were also infected with wild-type ATS2021 or ATS2021 *bimA_Bp_*. Figure 1D shows that wild-type ATS2021 also exhibited greater replication and survival than ATS2021 *bimA_Bp_* in C8-D1A cells at the 8 and 10 h post-infection time points.

**Fig 2:**
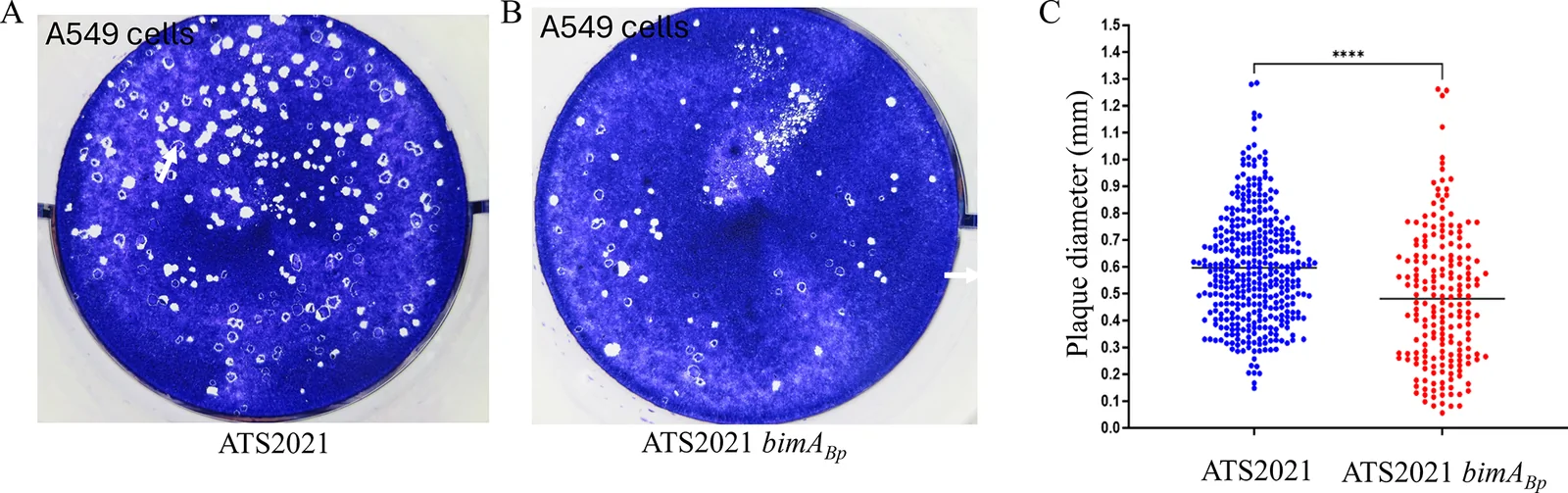
The *bimA_Bp_* mutant displayed altered intracellular pathogenesis *in vitro*. Plaque size comparison in host cells infected with *B. pseudomallei* ATS2021 and ATS2021 *bimA_Bp_* was assessed in A549 cells infected at an MOI of 1 with *B. pseudomallei* ATS2021 (**A**) and ATS2021 *bimA_Bp_* (**B**), and plaque formation was visualized by crystal violet staining 24 h after infection. Plaque diameters were determined with ImageJ software (**C**). At least 190 plaques were measured for each strain, and the difference between the median plaque size, shown as a horizontal black line, for each strain was analyzed with GraphPad Prism using a nonparametric *t*-test (Mann-Whitney *U* test). ****, *P* < 0.0001.

The human lung A549 cell line was infected with ATS2021 or ATS2021 *bimA_Bp_*, and both strains were able to form bacterial plaques at 24 h post-infection (Fig. 2A and B), demonstrating direct cell-to-cell spread in a nonphagocytic cell line. Interestingly, the median ATS2021 plaque diameter (0.58 mm) was significantly larger (*P* < 0.0001) than the 0.46 mm plaque diameter of ATS2021 *bimA_Bp_* (Fig. 2C). Taken together, these data indicate that *bimA_Bm_* and *bimA_Bp_* can both mediate actin-based motility in the isogenic ATS2021 background but suggest that the *bimA_Bm_* allele facilitates elevated levels of replication and cell-to-cell spread in a variety of cell types.

### The ATS2021 *bimA_Bp_* strain does not have a growth defect when compared to the wild-type ATS2021 strain but does have a defect in biofilm production *in vitro*

**Fig 3:**
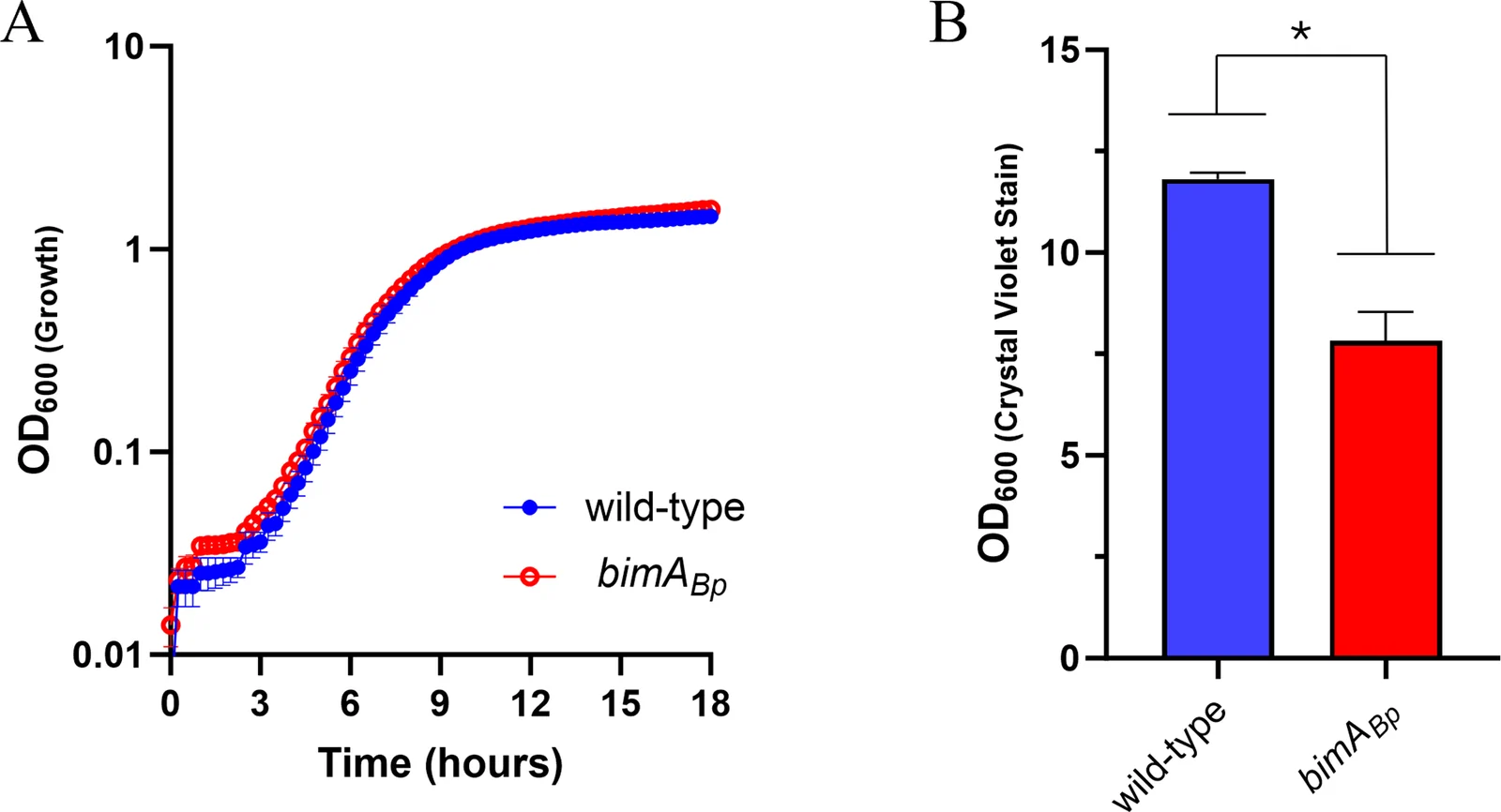
Growth and biofilm assessment of the *bimA_Bp_* mutant. Growth analysis was performed in LBG medium, measuring the OD_600_ with shaking at 37°C (**A**). Biofilm was quantified after static incubation at 37°C in LBG for 24 h (**B**). Error bars represent the standard error of the mean from three independent experiments. For biofilm data, pairwise treatment groups were compared by a linear mixed effects model. No multiplicity adjustment was applied. *, *P* < 0.033.

To assess if replacement of the native *bimA_Bm_* with the *bimA_Bp_* allele affected growth in liquid medium, growth curve analyses in rich medium (either GTB or LBG) at 37°C were performed. Under these conditions, no difference in planktonic growth was observed (Fig. 3A and data not shown). Previous reports indicate no growth rate differences in *B. pseudomallei* K96243 with a deletion of the *bimA* allele (36). We previously demonstrated that ATS2021 forms a robust biofilm in nutrient-replete conditions at 37°C; therefore, biofilm formation was also assessed with the ATS2021 *bimA_Bp_* strain (3). Unexpectedly, these experiments revealed that the *bimA_Bp_* variant exhibited a significant (*P* = 0.033) decrease in biofilm production relative to wild-type ATS2021 (Fig. 3B).

### The ATS2021 *bimA_Bp_* strain is significantly attenuated when compared to the wild-type ATS2021 strain in C57BL/6 mice

**Fig 4:**
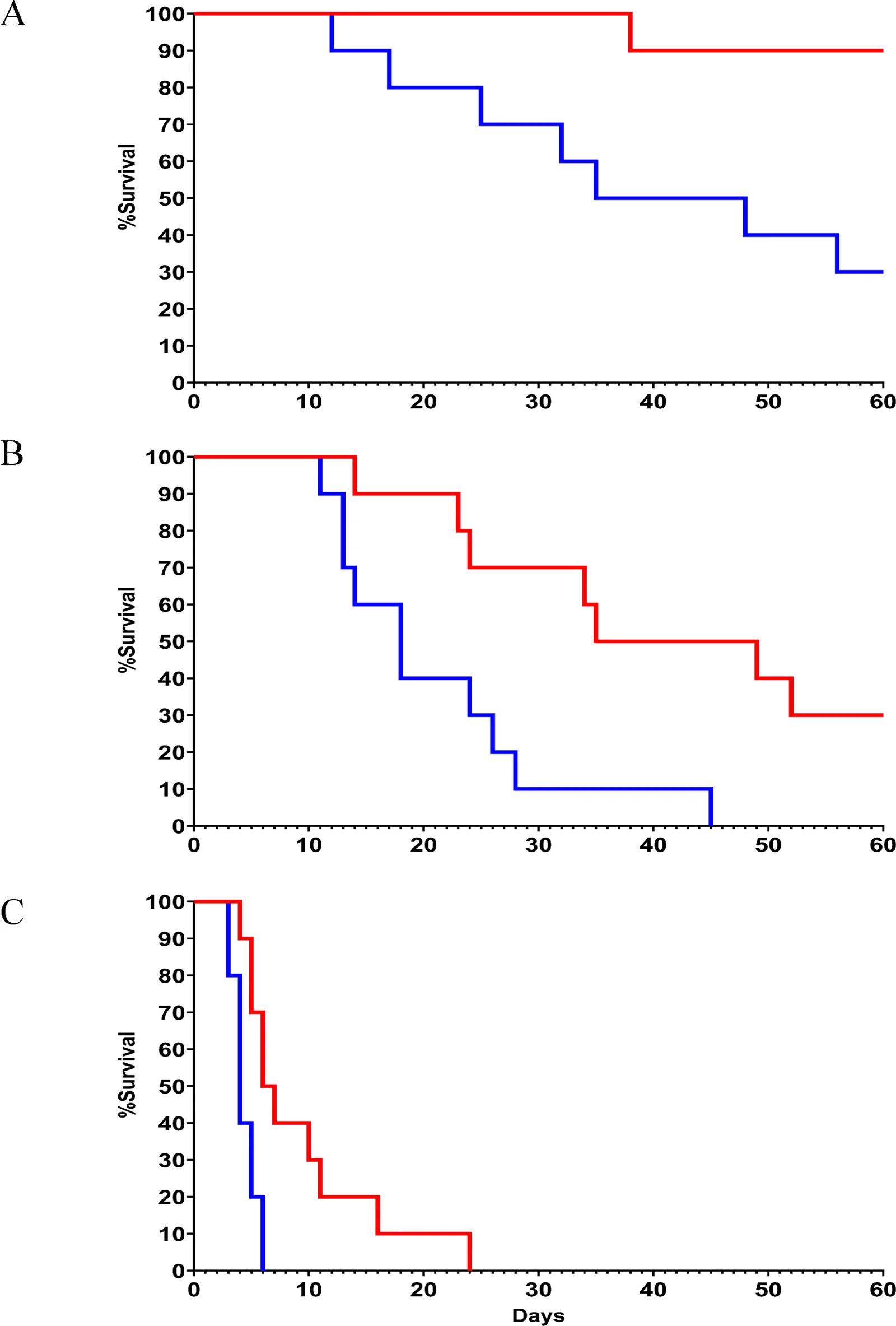
Determination of *in vivo* virulence of the *bimA_Bp_* mutant. Survival rates of female C57BL/6 mice exposed to aerosolized *B. pseudomallei* ATS2021 (blue lines) or ATS2021 *bimA_Bp_* mutant bacteria (red lines). Mice were exposed to 13 CFU of aerosolized ATS2021 or 23 CFU of aerosolized ATS2021 *bimA_Bp_*. Survival rates on day 60 and survival time on day 60 were both significantly different (*P* = 0.0198 and 0.0057, respectively) (**A**). Mice were exposed to 107 CFU of aerosolized ATS2021 or 204 CFU of aerosolized ATS2021 *bimA_Bp_*. Survival times on both days 21 and 60 were significantly different (*P* = 0.025 and 0.0044, respectively) (**B**). Mice were exposed to 1,149 CFU of aerosolized ATS2021 or 1,563 CFU of aerosolized ATS2021 *bimA_Bp_*. Survival times on day 21 were significantly different (*P* = 0.0032) (**C**). The survival rates at selected time points were compared by Fisher’s exact test.

For the wild-type ATS2021 strain of *B. pseudomallei*, a median lethal dose by aerosolization was previously calculated to be 56.4 CFU (95% confidence interval: 21.3, 150.2) at 21 days post-exposure and 5.8 CFU (95% confidence interval: 2.0, 15.1) for a 60-day duration post-exposure (3). To determine if the change to *bimA* affected the virulence of ATS2021, mice were challenged with aerosolized ATS2021 *bimA_Bp_* at a range of doses. The same target doses were used as in our earlier study on ATS2021 (1, 10, 10^2^, 10^3^, and 10^4^ CFU); actual inhaled doses were quantified as 5 CFU, 23 CFU, 204 CFU, 1,563 CFU, and 15,566 CFU (Table 1). Survival rates were determined on days 21 and 60 post-exposure (Table 2 and Fig. 4). The calculated LD_50_ for the ATS2021 *bimA_Bp_* strain was shown to be approximately 564.1 CFU (95% confidence interval: 271.5, 1,173.0) at 21 days post-exposure and 104.5 CFU (95% confidence interval: 46.5, 246.5) at 60 days post-exposure (Table 2). These differences were statistically significant for both the 21-day (*P* = 0.0016) and 60-day (*P* = 0.0002) survival analyses.

**TABLE 1:** Target doses and actual inhaled doses achieved for each bacterial strain

Target dose (CFU)	Inhaled wild-type ATS2021 (CFU)	Inhaled *bimA_Bp_* ATS2021 (CFU)
1	1	5
10^2^	13	23
10^3^	107	204
10^4^	1,149	1,563
10^5^	4,488	15,666

**TABLE 2:** LD_50_ calculation and descriptive statistics^*a*^

Day	Statistic and inhaled doses	ATS2021 *bimA_Bp_*	TTD median (95% CL)	TTD mean (standard error)
21	LD_50_ (95% CL)	564.1 (271.5, 1,173.0)		
	LD_50_ vs ATS2021 wild-type (*P*-value)	0.0016		
	5.09 CFU		>21	>21
	22.6 CFU		>21	>21
	204 CFU		>21	13.0 (.)
	1,563 CFU		6.5 (4.0, 11.0)	8.6 (1.4)
	15,666 CFU		4.0 (3.0, 4.0)	3.8 (0.1)
60	LD_50_ (95% CL)	104.5 (46.5, 246.5)		
	LD_50_ vs ATS2021 wild-type (*P*-value)	0.0002		
	5.09 CFU		>60	>60
	22.6 CFU		>60	38.0 (.)
	204 CFU		42.5 (13.0, .)	38.7 (4.8)
	1,563 CFU		6.5 (4.0, 11.0)	9.4 (2.0)
	15,566 CFU		4.0 (3.0, 4.0)	3.8 (0.1)

^*a*^ . indicates the value is not estimable.

As depicted in Fig. 4, there were noted significant differences between strains in the three target dose groups (10, 10^2^, and 10^3^ CFU) that we focused on. The differences observed in the 10 CFU target doses depicted in Fig. 4A were statistically significant for both survival rates (*P* < 0.02) and survival time (time to death from disease or euthanasia) through 60 days (*P* < 0.006). The differences in the 10^2^ and 10^3^ CFU target doses depicted in Fig. 4B and C did not demonstrate significantly different survival rates. However, with either target dose of the mutant *bimA_Bp_* bacteria, we observed extended survival times when compared to the mice infected with comparable doses of wild-type ATS2021 bacteria (*P* ≤ 0.025 for survival time calculated at days 21 and 60).

### Bacterial burden in mice post-exposure to aerosolized ATS2021 *bimA_Bp_* is lower when compared to that observed in mice infected with wild-type ATS2021

**Fig 5:**
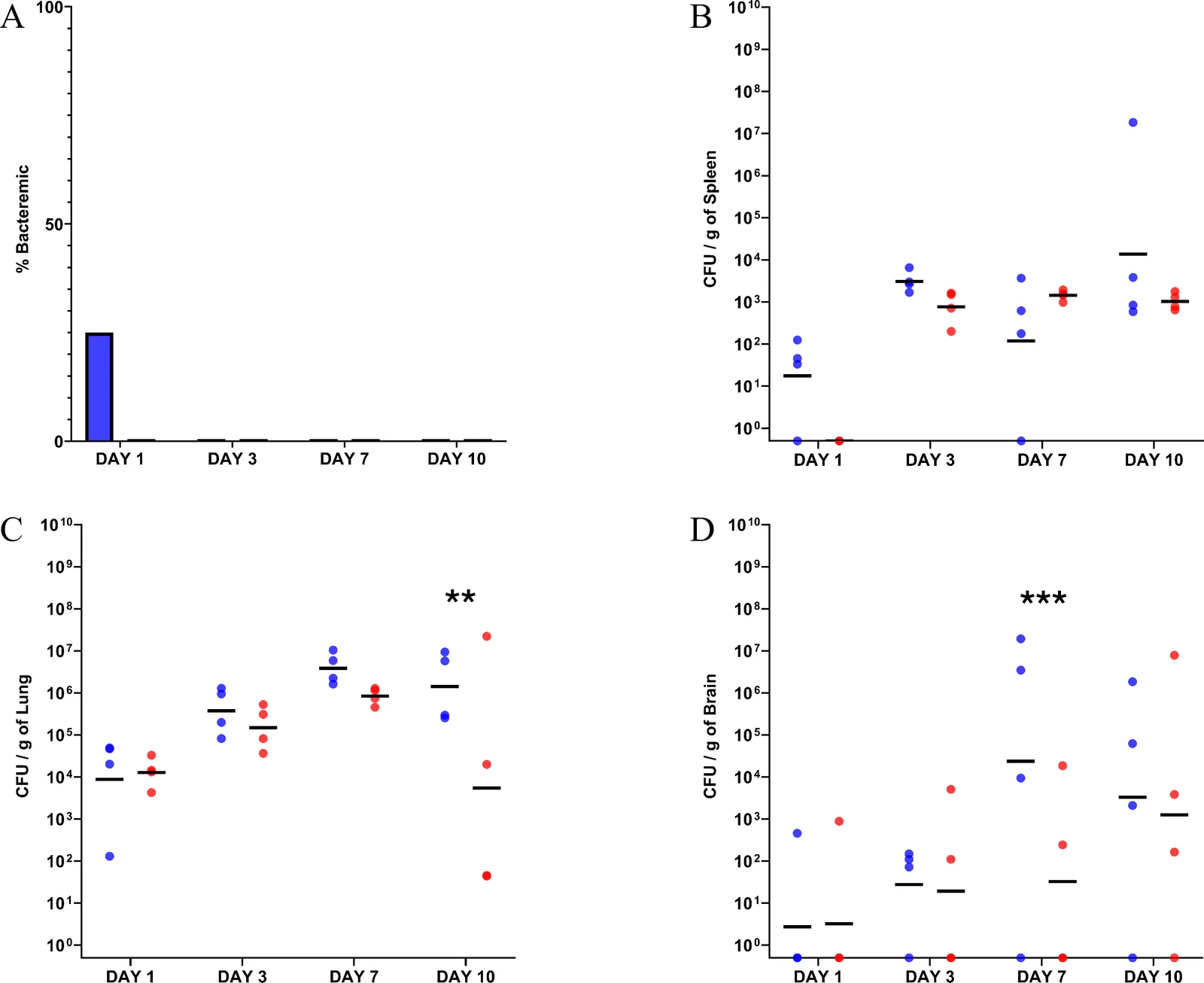
Evaluation of bacterial burden in select tissues 3 days post-exposure to a low dose of aerosolized *B. pseudomallei*. Mice were exposed to the 10^2^ CFU target dose and 204 CFU of aerosolized ATS 2021 *bimA_Bp_* (red). Mice were then serially sampled on days 1, 3, 7, and 10 to determine the percentage of mice that were bacteremic at that time point (**A**), bacterial burdens in spleen (**B**), lungs (**C**), and brains (**D**). These bacterial burdens were compared to previously published data detailing the bacterial burden after exposure to 107 CFU of aerosolized wild-type ATS2021 strain (blue). Representative histopathology in the brain of mice infected with this dose of bacteria is depicted in . For CFU data, pairwise treatment groups were compared using a linear mixed effects model. No multiplicity adjustment was applied. **, *P* < 0.005; ***, *P* ≤ 0.0005.

**Fig 6:**
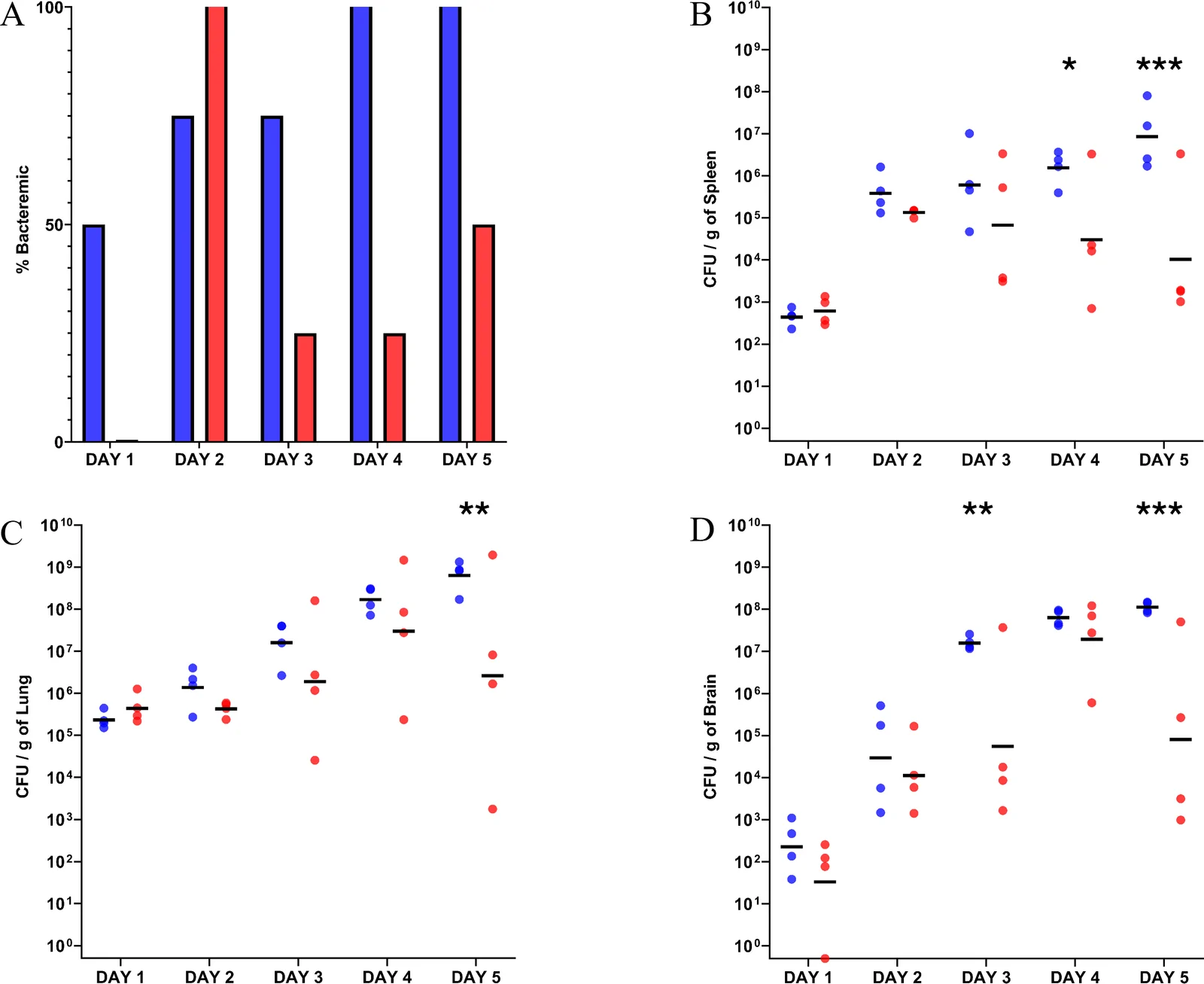
Evaluation of bacterial burden in select tissues 3 days post-exposure to a mid-dose of aerosolized *B. pseudomallei*. Mice were exposed to the 10^3^ CFU target dose and 1,563 CFU of aerosolized ATS 2021 *bimA_Bp_* (red). Mice were then serially sampled on days 1, 2, 3, 4, and 5 to determine the percentage of mice that were bacteremic at that time point (**A**), bacterial burdens in spleen (**B**), lungs (**C**), and brains (**D**). These bacterial burdens were compared to previously published data detailing the bacterial burden after exposure to 1,149 CFU of aerosolized CFU of the wild-type ATS2021 strain (blue). Representative histopathology in the brain of mice infected with this dose of bacteria is depicted in . For CFU data, pairwise treatment groups were compared using a linear mixed effects model. No multiplicity adjustment was applied. *, *P* < 0.05; **, *P* < 0.005; and ***, *P* ≤ 0.0005.

Three target dose groups (10^2^, 10^3^, and 10^4^ CFU) of aerosolized bacteria (parent vs variant) were evaluated for any potential differences in bacterial dissemination associated with the *bimA_Bp_* allele mutation. At the 10 CFU target dose, only one out of the four mice sampled exhibited bacteremia, and dissemination rates to the spleen were fairly comparable between the two strains of bacteria (Fig. 5). Interestingly, despite the higher inhaled dose of the mutant strain, the bacterial burden in the lungs (Fig. 5C) and brain (Fig. 5D) trended lower when the mice were infected with the ATS2021 *bimA_Bp_* strain, and the differences observed in the lungs at day 10 and the brains on day 7 reached statistical significance (*P* = 0.0025 and 0.0005, respectively). The 10^3^ CFU target dose (Fig. 6) displayed more significant differences in bacterial dissemination patterns. Overall, fewer mice were bacteremic at the days we examined when infected with the *bimA_Bp_* mutant strain (Fig. 6A). The bacterial burden in the spleen in mice exposed to the mid-dose range was virtually identical within 24 h after exposure to aerosolized bacteria, but within 72 h, the bacterial burden was lower in the mice that inhaled the *bimA_Bp_* mutant bacteria (Fig. 6B). These differences noted in the spleen were statistically significant on day 4 (*P* = 0.032) and day 5 (*P* = 0.0003). Similar dissemination kinetics were observed in the lungs (Fig. 6C) and the brains (Fig. 6D), indicating that despite having a higher inhaled dose, the mutant bacteria either did not replicate as well or survive as long in these organs. The differences noted in the lungs were significant on day 5 (*P* = 0.0029), and the differences in the brains were significant on days 3 and 5 (*P* = 0.0022 and *P* < 0.0001, respectively). Finally, at the 10^4^ CFU target dose, there were no statistically significant differences in bacterial burden within tissues between strains. It is important to note that, because of the high inhaled dose, mice could only be evaluated up to 72 h post-infection before succumbing or meeting euthanasia criteria. Additionally, the inhaled dose of the *bimA_Bp_* mutant strain was approximately three times higher than that of the wild-type ATS2021, likely further masking any potential differences in bacterial dissemination or replication at this dose range.

### Histological analyses of tissues from mice exposed to aerosolized ATS2021 *bimA_Bp_* bacteria

Histopathologic evidence consistent with *B. pseudomallei* was noted in many of the tissues examined from mice exposed to the 10^2^ or 10^3^ CFU target doses of the ATS2021 *bimA_Bp_* mutant bacteria. Following exposure to aerosolized *bimA_Bp_* mutant strain, histopathological changes were observed in the nasal turbinates, the olfactory nerves, the brain olfactory bulb, the lung, the liver, and the spleen. Few changes were noted in the cerebrum, the cerebellum, and the vertebral bone marrow, and these changes were only seen in the 10^3^ CFU target dose group of the ATS2021 *bimA_Bp_*-exposed mice (the higher of the two dose groups that we examined in histopathological analyses). Histological changes in these described tissues consisted of neutrophilic or suppurative inflammation, tissue necrosis with abundant necrotic debris, and hemorrhage. These characteristic lesions in tissues were evaluated for severity and scored and compared between two strains following exposure to 10^2^ or 10^3^ CFU target doses (Table 3). The highest scoring tissue lesions, following exposure to the 10^2^ CFU target challenge dose, were present in the lungs and the nasal turbinates. These were followed by the olfactory nerves and liver. Less prominent changes were present in the olfactory bulb, cerebrum, cerebellum, brain stem, spleen, and vertebral bone marrow. Importantly, exposure to the 10^2^ CFU target dose produced only minimal changes in the olfactory bulb, and there were no lesions in the cerebrum, cerebellum, or vertebral bone marrow. No lesions were noted in the spinal cord of any mice exposed to the *bimA_Bp_* variant. Representative images of the histopathological findings are shown in Fig. S2 and S3.

**TABLE 3:** Summary of top 10 most upregulated and downregulated genes found in mouse brains for days 3-5 following exposure to the aerosolized 10^3^ CFU target dose of wild-type ATS2021 (*bimA*_*Bm*_)

	*bimA*_*Bm*_ 1,000 CFU target dose								
	3 days post-challenge			4 days post-challenge			5 days post-challenge		
	Gene	Log2 Dif	*P*-value	Gene	Log2 Dif	*P*-value	Gene	Log2 Dif	*P*-value
Upregulated	Lcn2	11.3158	0.00013	Lcn2	12.0773	1.22E-09	Ccl2	12.8643	2.73E-06
	Ccl2	10.3424	0.001011	Ccl2	11.3886	1.20E-07	Lcn2	12.4511	2.52E-07
	Cxcl10	9.50086	0.000201	Cxcl10	9.13841	7.21E-08	Cxcl10	10.5347	1.73E-06
	Il1rn	8.30948	1.75E-05	Timp1	9.03727	3.90E-07	Timp1	9.99265	1.28E-05
	Ptx3	8.11612	0.002635	Ptx3	8.6243	5.12E-08	Ccl7	9.34928	2.06E-05
	Timp1	7.75208	0.000742	Ccl7	8.16616	1.69E-06	Tgm1	8.68299	3.60E-05
	Ccl7	7.1595	0.001729	Tgm1	8.08215	6.67E-06	Ptx3	8.63934	3.20E-06
	Il1b	6.8809	0.000145	Il1rn	7.61839	1.08E-05	Il1a	8.57013	1.22E-05
	Ccl3	6.75901	0.004903	Il1b	7.60177	5.16E-06	Il1b	8.4023	2.92E-05
	Il1r2	6.66391	0.000249	Il1a	7.32343	9.69E-07	Ccl3	8.26776	1.73E-06
Downregulated	Gpr34	−6.41088	0.000377	Gpr34	−6.72198	1.13E-15	Gpr34	−6.9759	7.63E-11
	Fcrls	−4.51658	0.000117	Fcrls	−4.88123	0.000206	Fcrls	−5.62666	4.92E-06
	P2ry12	−3.44773	0.00042	Ago4	−4.61857	1.16E-06	Rad51c	−5.51856	1.10E-08
	Slc2a5	−2.88042	0.017537	Ninj2	−3.96396	7.42E-11	Pllp	−5.29274	0.001695
	Ninj2	−2.86507	0.001852	P2ry12	−3.49304	0.000543	Enpp6	−5.20102	0.000132
	Hpgds	−2.63559	0.007303	Pllp	−3.44931	0.000511	Ago4	−4.87689	5.98E-05
	Pla2g5	−2.48308	0.005748	Enpp6	−3.16227	9.99E-06	Slc2a5	−4.31334	0.000337
	Adamts16	−2.32193	0.015675	Cd70	−3	0.004864	Ninj2	−4.21789	9.62E-08
	Pllp	−2.30933	0.001483	Pla2g5	−2.7885	0.002982	P2ry12	−3.77591	8.61E-05
	Cd70	−2.2854	0.032434	Slc2a5	−2.64386	0.003822	Chek2	−3.67243	0.003142

### Immunohistochemistry analyses of tissues from mice exposed to aerosolized ATS2021 *bimA_Bp_* bacteria

Immunohistochemistry using polyclonal rabbit anti-*Burkholderia* antibody was performed on head and spinal cord sections that included nasal turbinates, olfactory nerves, brain (olfactory bulb, cerebrum, cerebellum, and pons), and cervical, thoracic, and lumbar spinal cord, including the vertebral bone and bone marrow. Severity scores were used to indicate what percentage of the tissue section contained positive IHC labeling, and results are presented in Table 4 for the two different dose ranges we compared.

**TABLE 4:** Summary of top 10 most upregulated and downregulated genes found in mouse brains for days 3-5 following exposure to the aerosolized 10^3^ CFU target dose of mutant ATS2021 (*bimA*_*Bp*_)

	*bimA*_*Bp*_ 1,000 CFU target dose								
	3 days post-challenge			4 days post-challenge			5 days post-challenge		
	Gene	Log2 Dif	*P*-value	Gene	Log2 Dif	*P*-value	Gene	Log2 Dif	*P*-value
Upregulated	Lcn2	8.07146	0.000834	Lcn2	12.5529	5.52E-06	Cxcl10	9.96687	0.000262
	Cxcl10	7.08746	0.001758	Cxcl10	9.78856	5.18E-05	Ccl2	8.87581	0.000437
	Zbp1	6.40088	2.90E-05	Timp1	9.42154	0.000106	Lcn2	8.48119	0.008273
	Ccl5	5.62449	0.004391	Ccl2	8.88534	2.91E-05	Timp1	7.91588	0.001792
	Cxcl9	5.30986	0.009449	Cd14	8.507	0.000132	Il1a	7.80252	0.00177
	Gbp2	5.24447	0.001631	Il1b	8.46393	1.13E-05	Ccl7	7.56986	0.00191
	Rac2	4.9386	0.004427	Il1a	8.18115	0.000254	Cd14	6.89178	0.000949
	Igsf6	4.1964	0.011909	Il1rn	8.13827	9.65E-05	Serpine1	6.7252	0.004117
	Cd14	3.90689	0.020821	Serpine1	8.09878	0.000353	Cxcl9	6.35315	0.001244
	H2-T23	3.848	0.000253	Ccl7	8.04712	0.000106	Tgm1	6.21917	0.003906
Downregulated	Ninj2	−3.62449	0.012446	Gpr34	−3.18081	0.006789	Gpr183	−6.86831	0.004229
	Mapk12	−2.078	0.004165	Ninj2	−3.03526	0.003264	Top2a	−5.0287	0.010391
	Adamts16	−1.44057	0.030069	Igf1	−2.47073	0.013688	Grap	−4.91265	0.009191
	Blm	−1.40642	0.037429	P2ry12	−2.20914	0.01437	Cd6	−4.63904	0.016728
	Itga7	−1.1745	0.006588	Fcrls	−2.01886	0.019755	Myct1	−4.44207	0.014801
	Opalin	−0.958035	0.019142	Pllp	−1.69973	0.002018	Tnfrsf25	−4.42626	0.01377
	Gadd45a	−0.916651	0.004989	Slc2a5	−1.6845	0.023294	Cnn2	−4.30421	0.018843
	Tnfrsf12a	−0.863365	0.040377	Opalin	−1.40335	2.68E-05	P2ry12	−4.29778	0.010842
	Dock1	−0.662305	0.020415	Pla2g5	−1.30256	0.032734	Ezh2	−4.07003	0.019441
	Cdc7	0.595729	0.047501	Arc	−1.23429	0.001712	Itga6	−3.97218	0.01791

The highest IHC scoring animals corresponded to the 10^3^ CFU target challenge dose, and representative images are depicted in Fig. S4 and S5. The earliest IHC positivity was present in the nasal turbinates, olfactory nerves, and olfactory bulb by day 2 post-exposure in mice exposed to the 10^2^ CFU target dose of ATS2021 *bimA_Bp_* mutant. The highest IHC scoring tissues were the nasal turbinates and olfactory nerves (Table 4). Interestingly, there was no positivity observed in the olfactory bulb of mice after exposure to the 10^2^ CFU target dose of ATS2021 *bimA_Bp_* mutant (Table 4).

### Direct comparisons of histopathological and IHC analyses between wild-type ATS2021 and ATS2021 *bimA_Bp_* demonstrate clear differences attributed to the *bimA* allele

**Fig 7:**
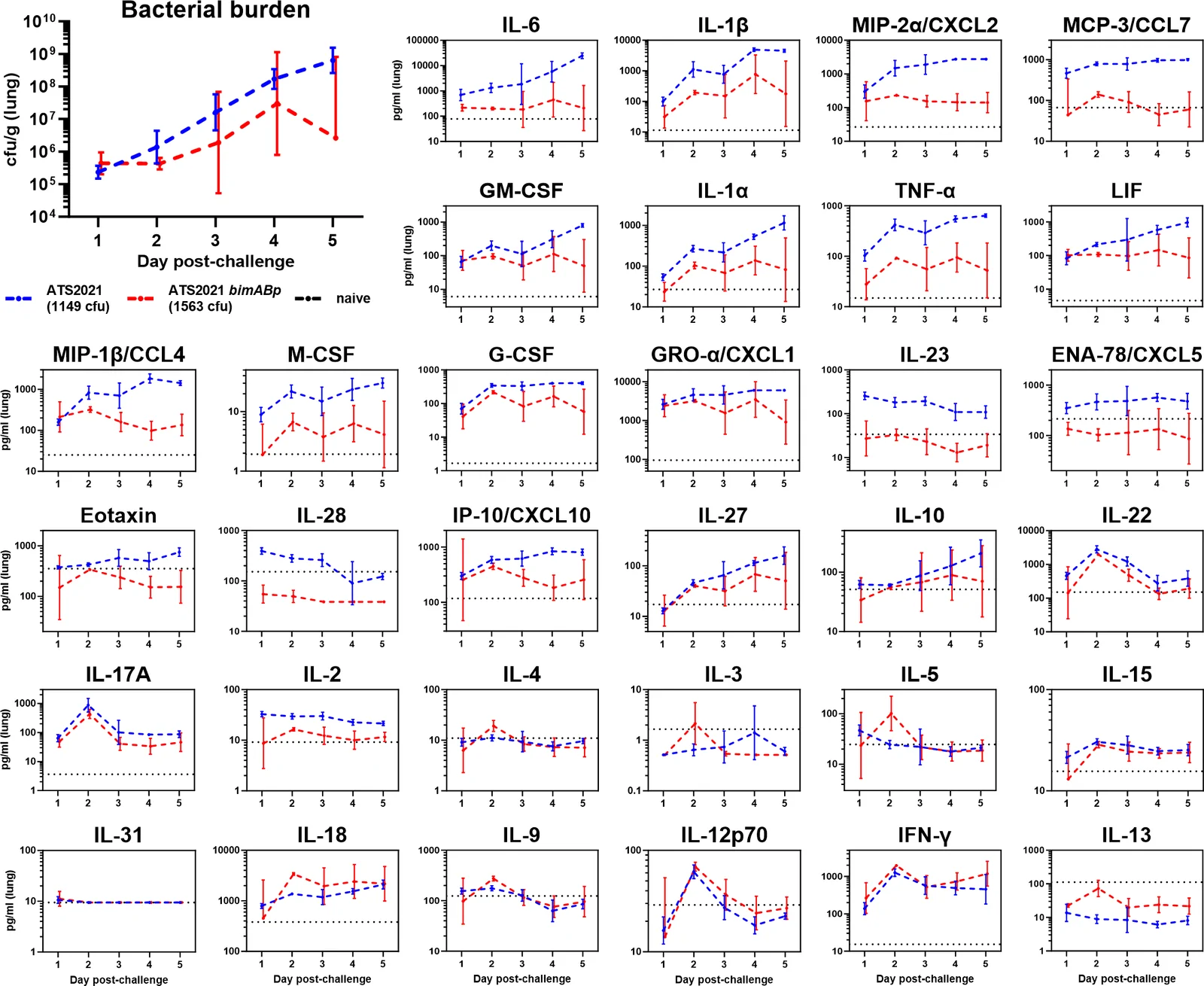
Evaluation of cytokine response in lung homogenates 3 days post-exposure to a mid-dose of aerosolized *B. pseudomallei*. Cytokine levels in lung homogenates from mice exposed to the 10^3^ CFU target dose; 1,149 CFU of aerosolized wild-type ATS2021 (blue) or 1,563 CFU of aerosolized ATS2021 *bimA_Bp_* (red) at days 1, 2, 3, 4, and 5 post-challenge (*n* = 4). Cytokines are sorted from the greatest difference (ATS2021 compared to ATS2021 *bimA_Bp_*) at day 5 post-challenge. Data are shown as geometric means, with error bars representing the geometric standard deviation. Black dotted line indicates the geometric mean of cytokine levels in naïve lung homogenates (*n* = 4). For Luminex data, pairwise treatment groups were compared by linear mixed effects model. No multiplicity adjustment was applied.

**Fig 8:**
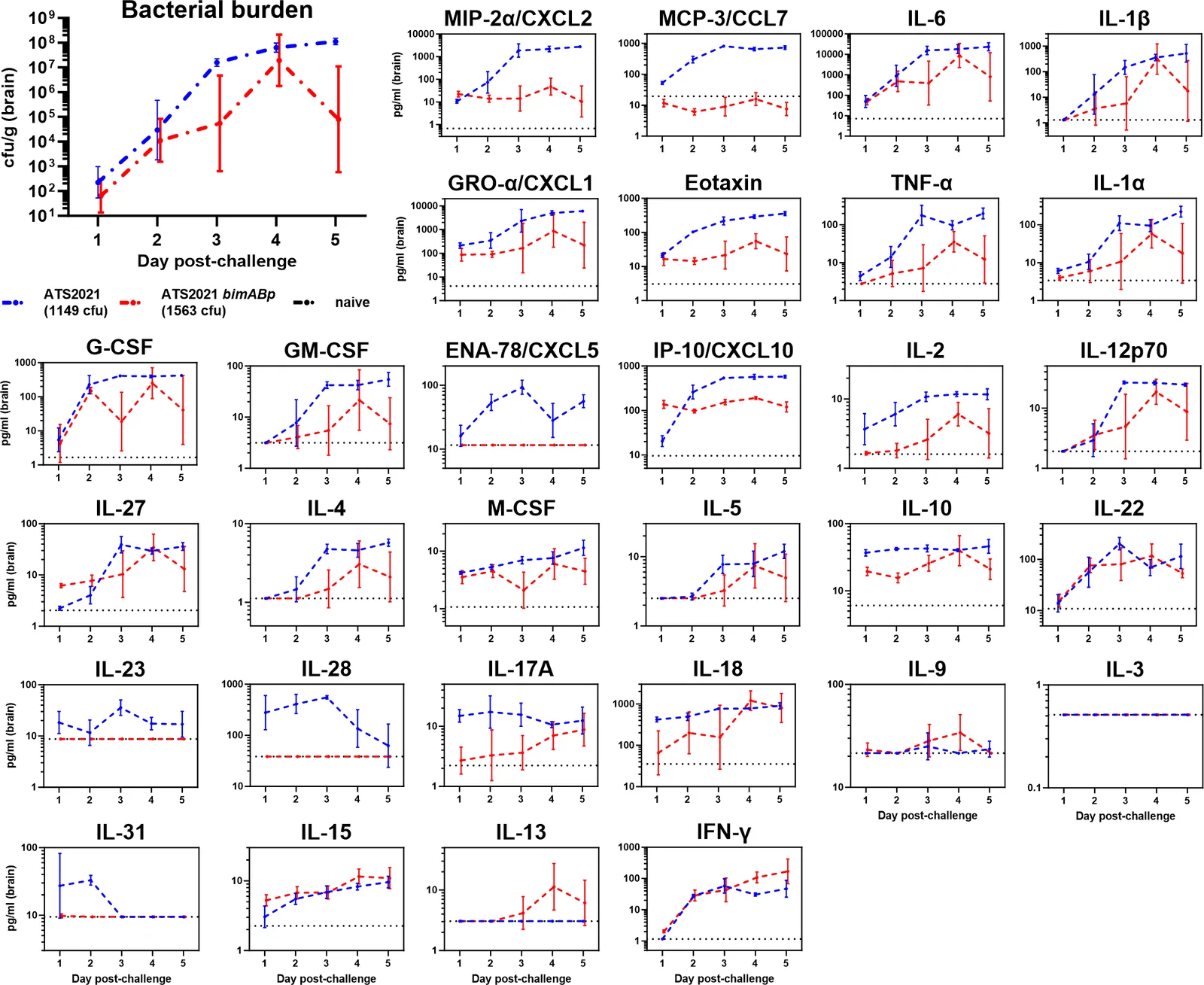
Evaluation of cytokine response in brain homogenates 3 days post-exposure to a mid-dose of aerosolized *B. pseudomallei*. Cytokine levels in brain homogenates from mice exposed to the 10^3^ CFU target dose; 1,149 CFU of aerosolized wild-type ATS2021 (blue) or 1,563 CFU of aerosolized ATS2021 *bimA_Bp_* (red) at days 1, 2, 3, 4, and 5 post-challenge (*n* = 4). Cytokines are sorted from the greatest difference (ATS2021 compared to ATS2021 *bimA_Bp_*) at day 5 post-challenge. Data are shown as geometric means with error bars representing the geometric standard deviation. Black dotted line indicates the geometric mean of cytokine levels in naïve brain homogenates (*n* = 4). For Luminex data, pairwise treatment groups were compared using a linear mixed effects model. No multiplicity adjustment was applied.

Compared to previously published pathological assessment of wild-type ATS2021, ATS2021 *bimA_Bp_* displays reduced virulence, diminished ability for neurologic invasion, and severely diminished ability to infect vertebral bone marrow (Fig. 7 and 8). Lesions in the lung and nasal turbinates were noted within 24 h post-exposure in mice exposed to the 10^3^ CFU target dose of ATS2021 *bimA_Bp_*, but were seen in both 10^2^ and 10^3^ CFU target doses of wild-type ATS2021. Minimal to moderate changes after exposure to 10^2^ CFU target dose of ATS2021 *bimA_Bp_* were not seen until day 3 post-exposure in the lung, liver, spleen, nasal turbinates, and olfactory nerves. Additionally, lesions in the cerebrum, cerebellum, and spinal cord were seen on days 6–10 post-exposure to 10^2^ CFU target dose of wild-type ATS2021 but were never seen after exposure to 10^2^ CFU target dose of ATS2021 *bimA_Bp_*.

When comparing 10^3^ CFU target dose groups by day 3 post-exposure, the wild-type challenged mice displayed mild to marked lesions in the lung, liver, spleen, nasal turbinates, olfactory nerves, olfactory bulb, and vertebral bone marrow, whereas those challenged with 10^3^ CFU target dose of ATS2021 *bimA_Bp_* displayed mild to marked lesions in the lung, liver, spleen, nasal turbinates, olfactory nerves, and olfactory bulb. Additionally, 10^3^ CFU target challenge dose with wild-type ATS2021 caused bone marrow osteomyelitis in all animals starting at day 3 post-exposure to aerosolized bacteria. This contrasts with the mice receiving the 10^3^ CFU target dose of the mutant *bimA_Bp_* bacteria, which only caused minimal to mild bone marrow lesions on day 5 in two animals.

Overall, the total severity score of histological lesions for these animals is higher for wild-type ATS2021 (scores of 231 for mice challenged with 10^2^ CFU target dose and 474 for mice challenged with 10^3^ CFU target dose) compared to the mutant ATS2021 *bimA_Bp_* (scores of 83 for mice challenged with 10^2^ CFU target doses and 260 for mice challenged with 10^3^ CFU target dose). Immunohistochemistry results support these findings when comparing total severity scores of all animals challenged with wild-type ATS2021 (scores of 112 for 10^2^ CFU target dose and 204 for 10^3^ CFU target dose), which exhibited more *Burkholderia* staining than the mice challenged with the ATS2021 *bimA_Bp_* (scores of 15 for 10^2^ CFU target doses and 151 for 10^3^ CFU target dose).

### Mice exposed to aerosolized wild-type ATS2021 *bimA_Bm_* or mutant ATS2021 *bimA_Bp_* display differing immune responses to the infections as determined by cytokine expression

**Fig 9:**
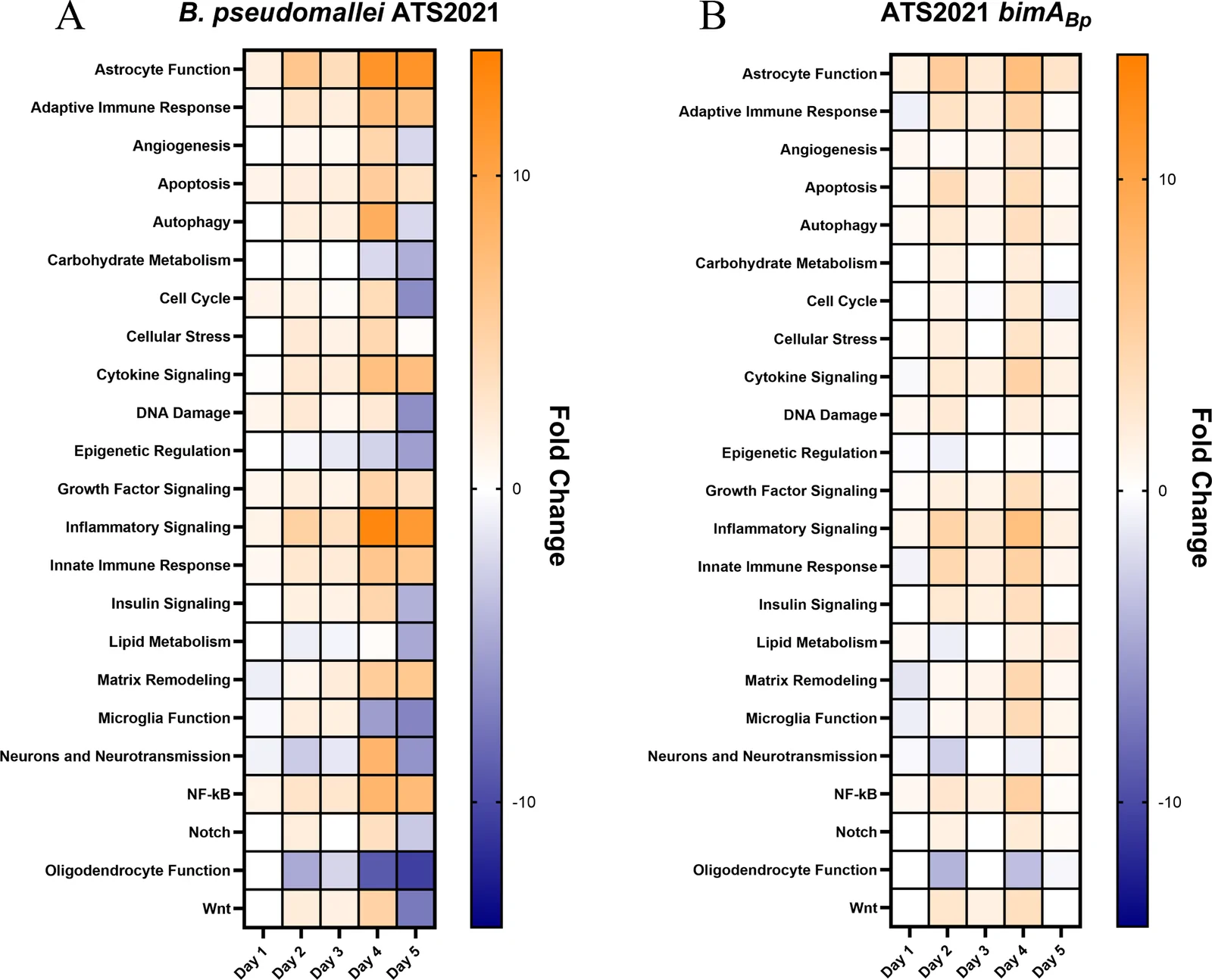
Host transcriptomic analyses in brain homogenates 3 days post-exposure to a mid-dose of aerosolized *B. pseudomallei*. Heat maps displaying directed gene set analysis enrichment scores derived from brain homogenates of mice aerosol challenged with the 10^3^ CFU target dose of either wild-type *B. pseudomallei* ATS2021 (**A**) or ATS2021 *bimA_Bp_* mutant (**B**). The first 5 days post-challenge are displayed, with heat map colors denoting relative positive (upregulated) or negative (downregulated) directional scores of 23 neuroinflammatory pathways represented in this panel.

Following exposure to the 10^3^ CFU target challenge, the prevailing pattern of the immune response in lung homogenates was characterized by increasing cytokine levels from day 1 to day 5 after ATS2021 challenge. In contrast, the cytokine levels peaked earlier after challenge with the *bimA_Bp_* mutant, leading to a considerable divergence between the two strains by day 5 (Fig. 9). This tracks the pattern of bacterial burden in the two strains, with the exception that bacterial burden of the *bimA_Bp_* mutant at day 2 was still at the same low level as day 1, while several cytokines peaked at day 2 of challenge with the mutant and were lower on days 3–5. These include CCL4, CCL7, CXCL2, CXCL10, and G-CSF. Several other cytokines plateau from day 2 to day 5 after challenge with the mutant (e.g., IL-6, IL-1α, LIF, and TNF-α) while continuing to rise after challenge with ATS2021.

On day 1 of the wild-type ATS2021 challenge, several cytokines were already expressed at significantly higher levels in lungs compared to challenge with the mutant, despite the slightly higher bacterial burden with the mutant strain (Table S1). These included IL-2, IL-10, IL-23, IL-28, CCL7, CXCL5, M-CSF, and TNF-α. IL-2 was consistently expressed above baseline with the wild-type ATS2021 challenge, but at or below baseline with the mutant. No cytokines showed the reverse pattern. However, on day 2 of the challenge, several cytokines were expressed at significantly higher levels in mice infected with the *bimA_Bp_* mutant than in mice infected with wild-type ATS2021 (IL-3, IL-4, IL-5, IL-9, IL-13, and IL-18). IFN-γ had a unique pattern of peaking at day 2 with both strains, then peaking again at day 5 with the *bimA_Bp_* mutant, while staying stable from day 3 to 5 with ATS2021.

In the 10^4^ CFU target dose group, all mice died or were euthanized by day 4. The bacterial burden in lungs was higher after challenge with the *bimA_Bp_* mutant than with ATS2021, which could be due to the *bimA_Bp_* mutant inoculum being threefold higher. Nonetheless, many cytokines were significantly higher in wild-type ATS2021-challenged lungs at most or all time points, including IL-2, IL-23, IL-28, CCL7, CXCL2, CXCL5, M-CSF, and TNF-α (Fig. S6 and Table S1). As in the 10^3^ CFU target dose group, IL-13, IL-18, and IFN-γ showed higher levels with the mutant than with ATS2021, significantly higher on at least one time point.

In the 10^2^ CFU target dose group, most lung cytokine levels were notably lower than in the higher two dose groups, especially proinflammatory cytokines, and there were fewer significant differences between groups (Fig. S7 and Table S1). Some cytokines were higher with wild-type ATS2021 challenge than with the *bimA_Bp_* mutant by day 7, around the peak of bacterial burden, before decreasing by day 10 (IL-1α, IL-23, IL-28, CCL7, M-CSF, and TNF-α), some of which were significantly higher at day 1. However, unlike with the higher challenge doses, at the 10^2^ CFU target dose, several cytokines were higher in mice challenged with the mutant, particularly on day 1 (IL-3, IL-4, IL-5, IL-12p70, CCL4, CXCL2, and CXCL10), indicating earlier upregulation of these factors with the *bimA_Bp_* allele.

**Fig 10:**
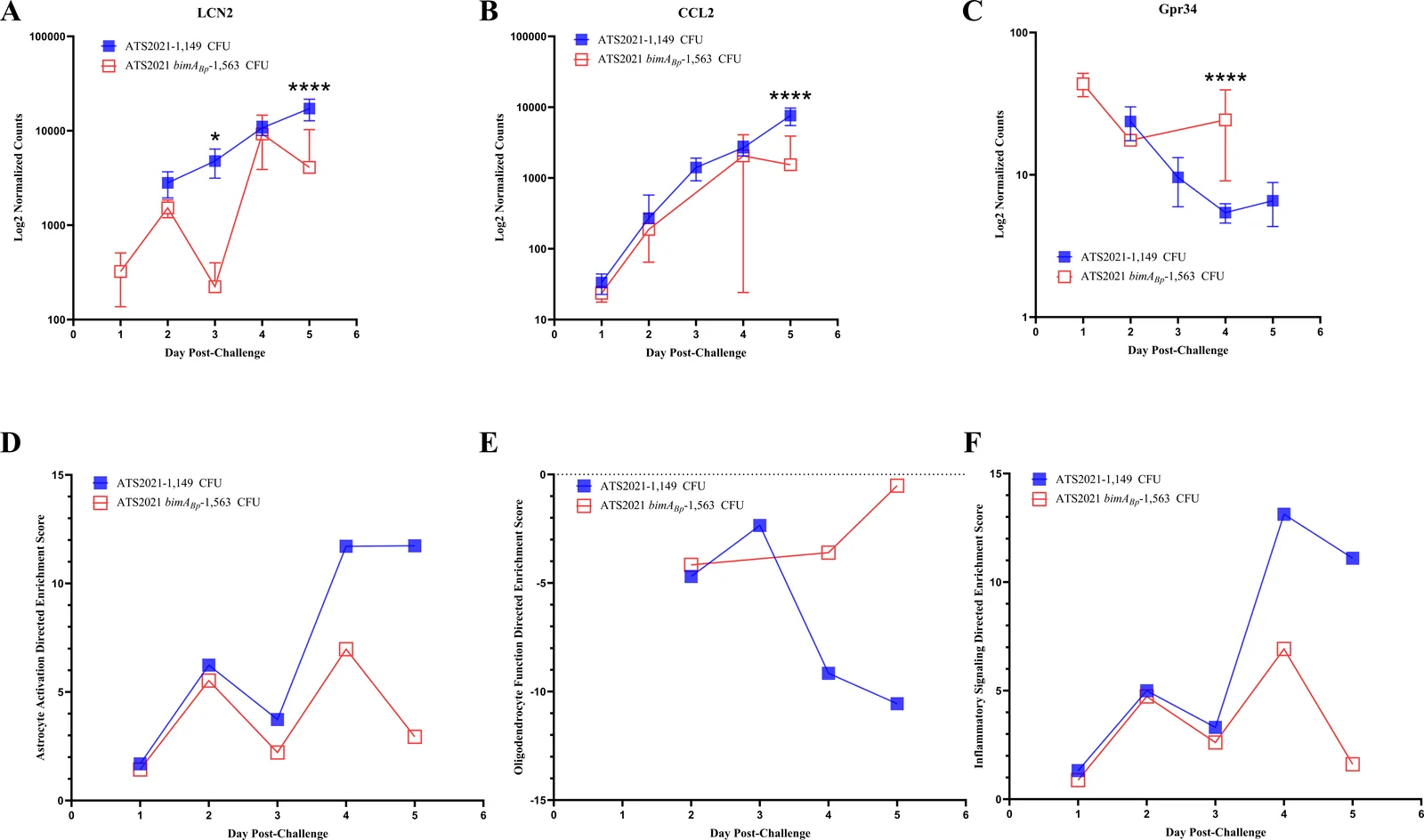
Expression of specific genes associated with neurological function in brain homogenates 3 days post-exposure to a mid-dose of aerosolized *B. pseudomallei*. Differences in enriched pathways and select genes observed in brains of mice aerosol challenged with the 10^3^ CFU target doses of (1,149 CFU) wild-type ATS2021 (blue) or (1,563 CFU) ATS2021 mutant *bimA_Bp_* (red). Expression changes over time in select genes associated with LCN2 (**A**), CCL2 (**B**), and Gpr34 (**C**) genes for the 10^3^ CFU target dose group. Directed global enrichment scores for astrocyte activation (**D**), oligodendrocyte function (**E**), and inflammatory signaling (**F**) observed for 5 days following aerosol challenge. Error bars represent standard deviation. *P ≤ 0.05; ****P ≤ 0.0001.

In naïve brain tissue, only 7/32 cytokines were detected above the lower limit of detection in our Luminex immunoassay (IL-10, IL-17A, CCL7, CXCL1, CXCL10, M-CSF, and Eotaxin), whereas in naïve lungs, this was 29/32 cytokines. In brain homogenates, following exposure to the 10^3^ CFU target dose challenge, the prevailing pattern was for cytokines to be at similar levels in the two strains on day 1 post-challenge, then diverging on day 2 as the cytokine levels increase with wild-type ATS2021 bacteria faster than with the *bimA_Bp_* mutant bacteria, and significantly different by day 3 (Fig. 10; Table S2), As in lung homogenates, the cytokine response in the brain roughly tracks the bacterial burden detected in the tissue, with levels in ATS2021 infection staying high through day 5, while levels in *bimA_Bp_* mutant infection peak on day 4 post-exposure.

In the 10^3^ CFU target dose group, the bacterial burden in the brain was higher with wild-type ATS2021 than the *bimA_Bp_* mutant at all five time points, even on day 1, despite the inoculum being approximately 36% higher with the *bimA_Bp_* mutant. However, on day 1, only a few cytokines were significantly higher with ATS2021 infection than the *bimA_Bp_* mutant (CCL7, CXCL5, IL-2, IL-10, IL-17A, IL-18, IL-23, and IL-28). Interestingly, these were mostly cytokines that defied the general pattern, following infection with the *bimA_Bp_* mutant strain, by having similar levels at all time points or even decreasing between days 1 and 5. For some of these, levels increased after day 1 with the mutant and converged with ATS2021 levels by day 4 or 5 (IL-10, IL-17A, and IL-18).

A few cytokines (IL-15, IL-27, and CXCL10) in the brain homogenates had higher levels on day 1 after challenge with the *bimA_Bp_* mutant, despite the higher day 1 bacterial burden with wild-type ATS2021 (Fig. 10). This expression profile for IL-27 and CXCL10 was also observed in the lungs of mice exposed to the 10^2^ CFU target dose of bacteria, suggesting that the strain with the *bimA_Bp_* allele induces early upregulation of these two cytokines before they peak at similarly high levels later in disease progression(Fig. S7). Furthermore, in both brains and lungs, the IFN-γ response was initially consistent between both strains, diverging at later time points, with higher levels after infection with the *bimA_Bp_* mutant compared with wild-type ATS2021.

The kinetics of bacterial burden in the brain after exposure to the 10^4^ CFU target dose challenge groups were different from the 10^3^ CFU target dose group; although ATS2021 had slightly higher bacterial burdens on day 1, by day 2 (and day 2.5), there were higher bacterial burdens with the *bimA_Bp_* mutant(Fig. S8). This suggests that the bacteria reached the brain faster with wild-type ATS2021, but the threefold higher inoculum of the mutant meant that, once it reached the brain, it was present in higher numbers. Comparing the cytokine response in the brain between 10^3^ and 10^4^ CFU target dose groups at the 2- and 3-day time points shared by both groups reveals that several cytokines (IL-1α, IL-4, GRO-α/CXCL1, GM-CSF, and TNF-α) that were higher in wild-type ATS2021 in the 10^3^ CFU target dose group were expressed at similarly high levels in both strains with 10^4^ CFU target doses (Fig. 10 and Fig. S8). Meanwhile, most of the cytokines that already displayed a significant difference between strains at day 1 in the 10^3^ CFU target dose groups showed the same pattern with the 10^4^ CFU target dose group (Table S2). For example, IL-2, IL-10, IL-17A, IL-18, IL-23, and IL-28 were higher at day 1 with wild-type ATS2021, while with the *bimA_Bp_* mutant, IL-27 and CXCL10 were initially higher, but IFN-γ and IL-13 were higher at later time points.

Cytokine responses in the brain after challenge with the 10^2^ CFU target dose group presented with greater variance in the data for both strains, possibly due to the detection of bacteria in only a subset of brains. Among mice challenged with wild-type ATS2021, the number with detectable CFU in the brain was 1/4 (day 1) and 3/4 (days 3, 7, and 10). Among mice challenged with the *bimA_Bp_* mutant, the number with detectable CFU in the brain was 1/4 (day 1), 2/4 (days 3 and 7), and 3/4 (day 10). As in both the 10^3^ and 10^4^ CFU target dose groups, most cytokine responses correlated with bacterial burden and were higher in wild-type ATS2021 on day 7, the only day that wild-type ATS2021 had a higher bacterial burden than the mutant. On day 3, several cytokines were higher after challenge with *bimA_Bp_* mutant than with wild-type ATS2021, although this was only significant for IL-15, IL-27, and IL-22 (Fig. S9 and Table S2).

On day 1 post-exposure, the trend seen in brains of mice challenged with 10^3^ or 10^4^ CFU target dose was recapitulated in mice infected with the lowest challenge dose; a few cytokines, including IL-2, IL-17A, IL-18, IL-23, and IL-28, were elevated at day 1 with wild-type ATS2021, and IL-27 and CXCL10 were elevated with the mutant, though IL-27 did not reach significance until day 2 (Fig. S9). For all these seven cytokines, at least three of four mice in the group had elevated levels by day 1 post-exposure, despite only one of four having detectable bacteria in the tissue.

### Mice exposed to aerosolized wild-type ATS2021 *bimA_Bm_* or mutant ATS2021 *bimA_Bp_* display differing immune responses to the infections in brains, as determined by host transcriptomic analyses

**Fig 11:**
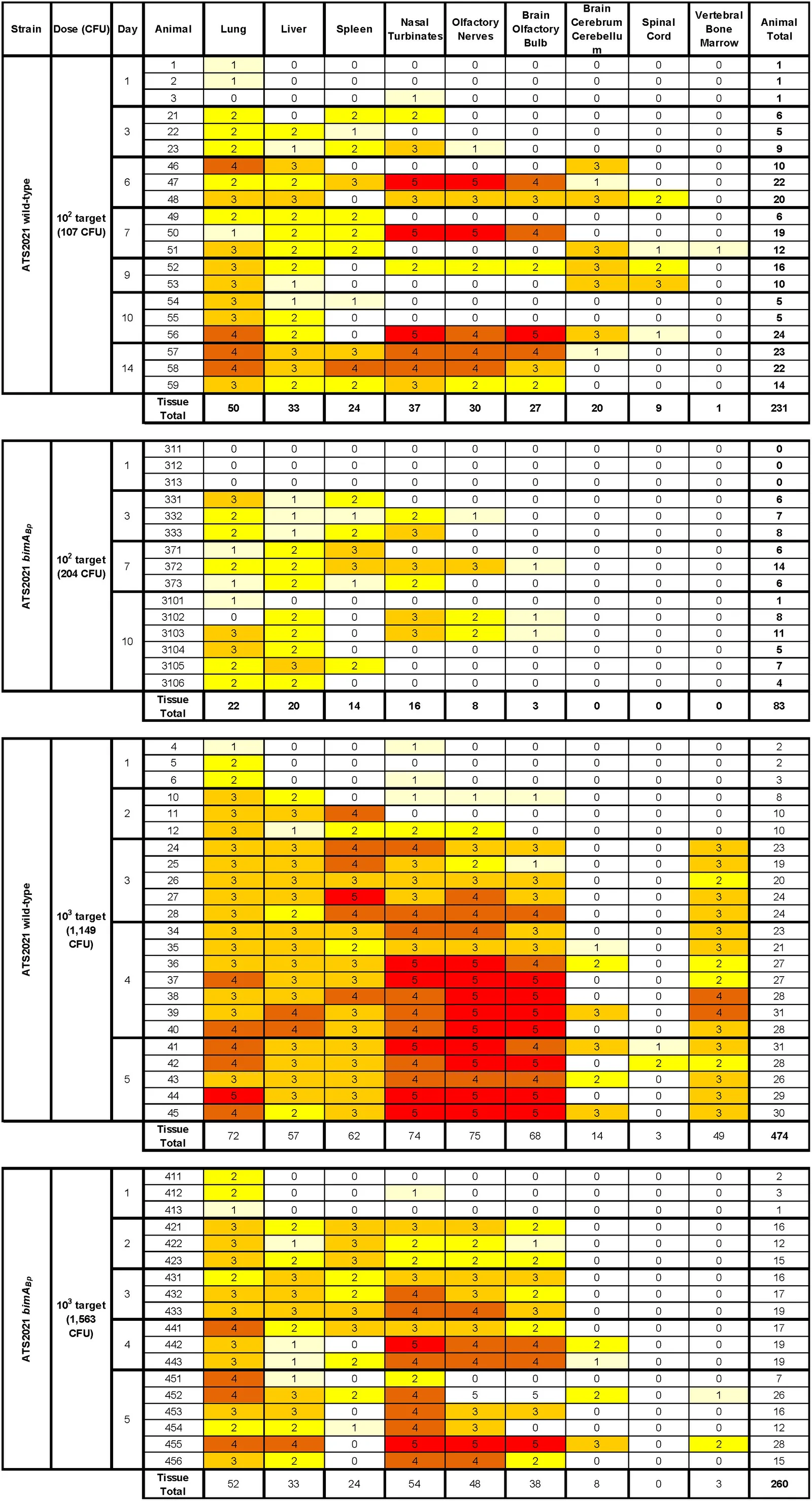
Histological comparison of mice exposed to 10^2^ and 10^3^ CFU target doses of wild-type ATS2021 and ATS2021 *bimA_Bp_.* Representative histopathology in the brains of mice infected with these doses of bacteria is depicted in Fig. S2 to S5. 0, none; 1, minimal 0%–10%; 2, mild 10%–25%; 3, moderate 25%–50%; 4, marked 50%–75%; and 5, severe 75%–100%.

**Fig 12:**
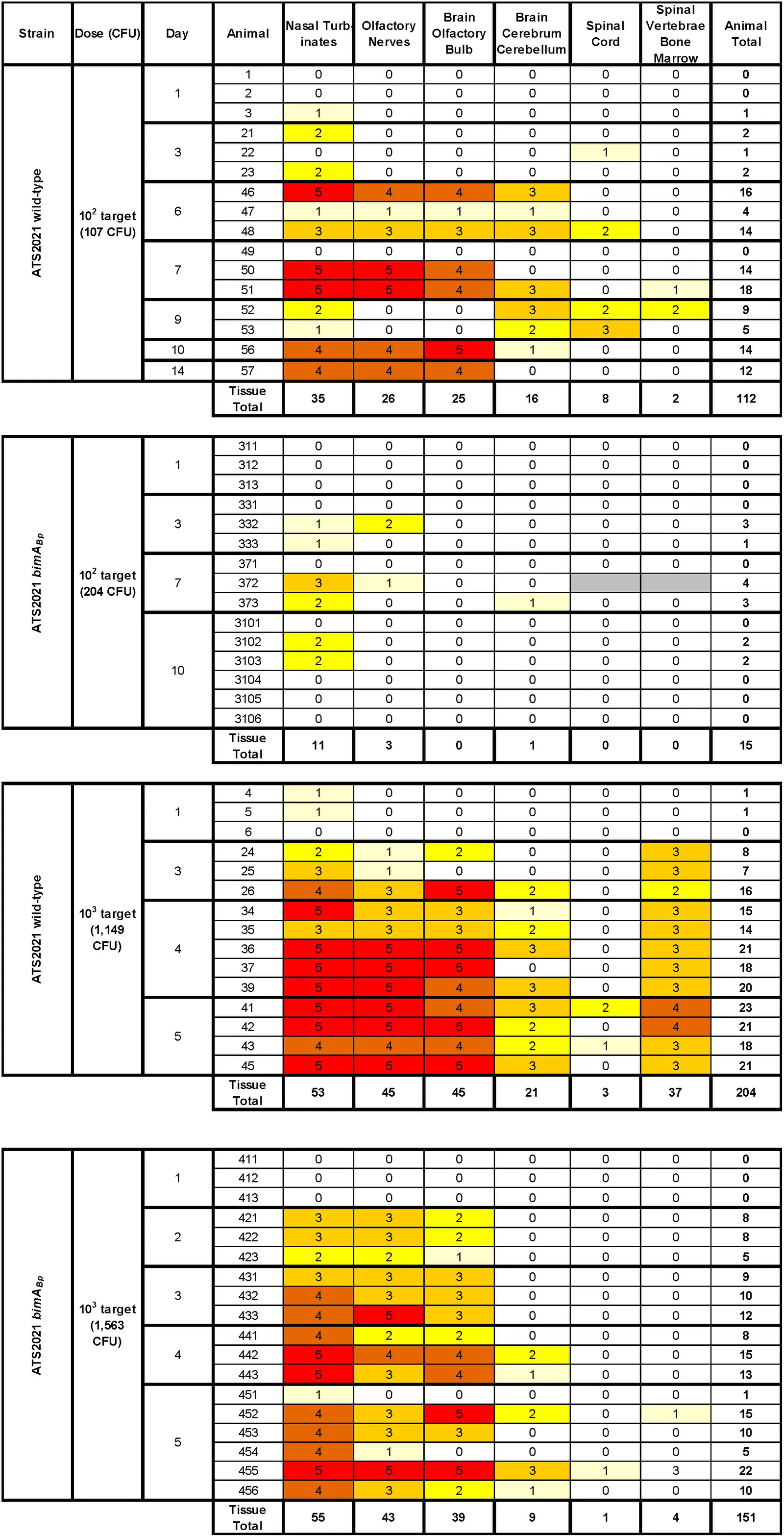
Immunohistochemical comparison of mice exposed to 10^2^ and 10^3^ CFU target doses of wild-type ATS2021 and ATS2021 *bimA_Bp_.* Representative histopathology in the brains of mice infected with these doses of bacteria is depicted in Fig. S2 to S5. 0, none; 1, minimal 0%–10%; 2, mild 10%–25%; 3, moderate 25%–50%; 4, marked 50%–75%; and 5, severe 75%–100%.

A targeted transcriptomics approach was employed to assess host gene expression changes following exposure to wild-type ATS2021 (*bimA_Bm_*) and mutant ATS2021 *bimA_Bp_* by analyzing total RNA extracts from mouse brain homogenates with a neuroinflammatory NanoString probe panel consisting of hundreds of genes associated with neuroinflammation, immune modulation, and homeostasis of various brain cell populations (Table S3). Widespread gene expression changes were observed in mice challenged with either isolate across a range of doses (Table S4 and Tables S5–S8). The most significant changes were consistently seen over time in the 10^3^ and 10^4^ CFU target dose groups, which also corresponded to the more acute disease phenotype. Relative expression difference observed between the two challenge strains at the 10^3^ CFU target-dose is shown in Fig. 11. For comparison of changes in gene transcription levels between the bacterial strains harboring the different *bimA_Bm_* and *bimA_Bp_* alleles, the 10^3^ CFU target dose groups were selected. Analysis of the most upregulated and downregulated differentially expressed genes between isolates identified several conserved genes that were modulated in ATS2021 strains independent of the *bimA* variant. Notably, both strains induced robust activation of inflammatory genes encoding Lipocalin2, CXCL10, and CCL2, while *Ninj2* and *Pllp*, genes associated with oligodendrocyte function, were downregulated. Importantly, robust differences were noted in both the magnitude of DEGs between these strains as well as the pathways they associate with. At 3 days post-exposure, conserved DEGs (i.e., *Lcn2*, *Ccl2*, and *Gpr34*) observed in the wild-type *bimA_Bm_* challenge group were generally at a higher (upregulated) or lower (downregulated) level of expression relative to the equivalent trend seen in the mutant *bimA_Bp_* challenge group (Tables 3 and 4 and Fig. 10A through C). Transcripts associated with three pathways/networks were identified, which were highly upregulated or downregulated across later time points: astrocyte activation, inflammatory signaling, and oligodendrocyte function, with the former two pathways/networks displaying a trend of higher scores for the wild-type ATS2021 relative to the *bimA_Bp_* mutant, while the oligodendrocyte function pathway/network was lower in the wild-type ATS2021 group than the *bimA_Bp_* mutant (Fig. 12D through F).

### Infection with wild-type ATS2021 results in a greater level of glial cell activation compared to mice infected with ATS2021 *bimA_Bp_*

**Fig 13:**
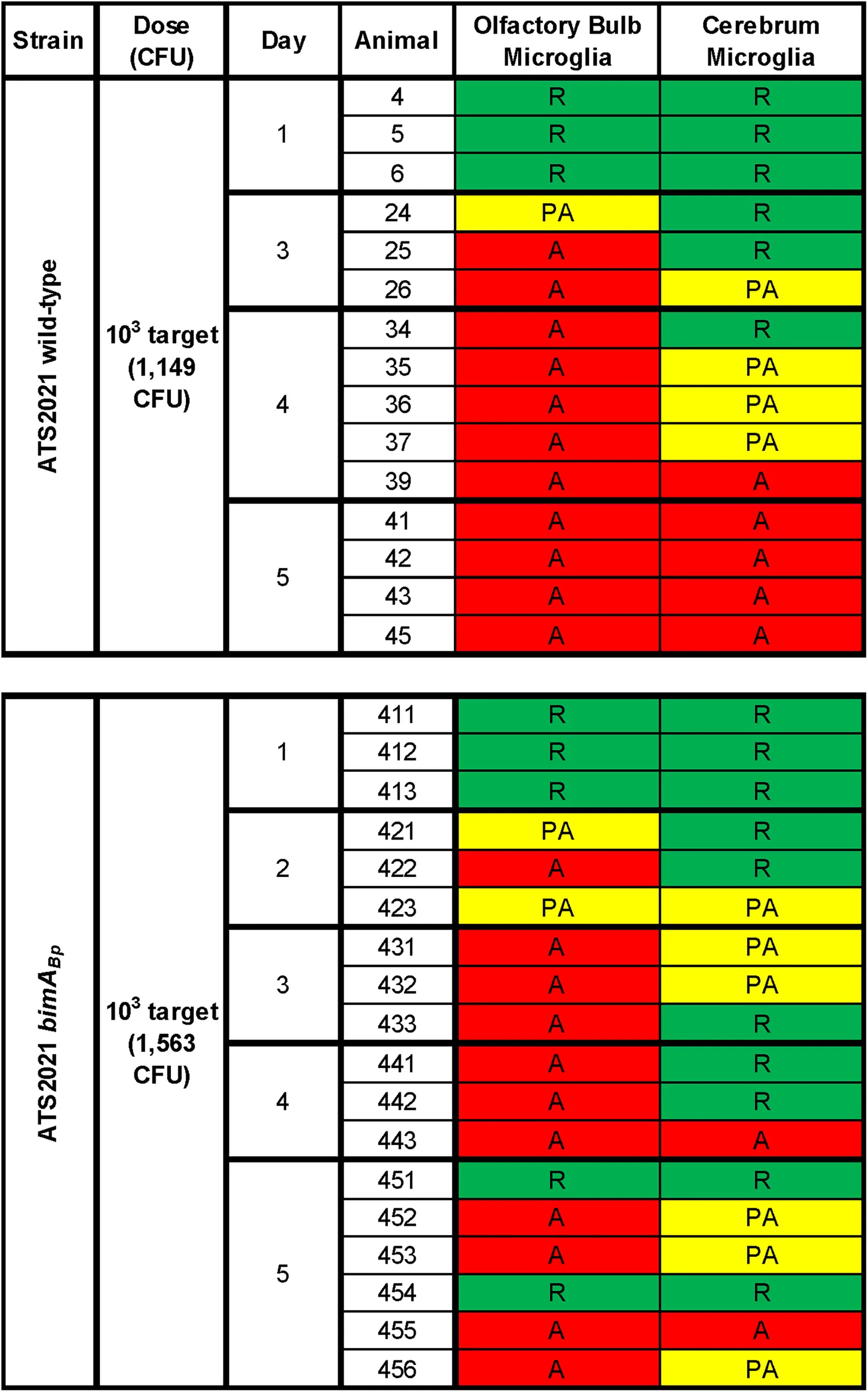
Comparison of glial cell activation as determined by IHC analyses of brain sections from mice exposed to the 10^3^ CFU target dose. A, some or many (25%–100%) microglia appear activated (bushy and/or amoeboid microglia); PA, few to some (1%–25%) of microglia appear activated; R, most or all (75%–100%) microglia appear ramified (ramified and/or hyper-ramified microglia).

Activated microglia produce CCL2 as part of the neuroinflammatory response and are the primary source of CCL2 in the brain. Microglial activation may be due to the presence of pathogens, cellular damage, and signaling molecules like TNF-α, IL-1, and IL-6. To investigate this further, we performed Iba1 immunohistochemistry on brain sections from mice exposed to the 10^3^ CFU target dose. Iba1 IHC is a marker for microglia and macrophages. With the Iba1 IHC, we were able to identify microglia that were quiescent (ramified or hyper-ramified) and partially or fully activated (bushy or amoeboid). As demonstrated in and Fig. 13, there was more evidence of microglia activation in the brains of mice exposed to aerosolized wild-type ATS2021 compared to the brains of mice exposed to aerosolized *bimA_Bp_* bacteria.

## DISCUSSION

This work identifies differences attributable to the *bimA* allele by characterizing an isogenic mutant constructed in the ATS2021 strain of *B. pseudomallei*. Replacing the *bimA_Bm_* allele with *bimA_Bp_* demonstrates that the *bimA_Bp_* allele is associated with significantly decreased replication or survival in several cell types, including astrocytes and macrophages, a significantly increased LD_50_ estimation when delivered as small particle aerosols to C57BL/6 mice, altered biofilm production, and significantly different bacterial dissemination patterns in multiple organs, including the brain, likely contributing to decreased overall virulence. While we are uncertain of the exact role of BimA in biofilm formation, we noted a significant decrease, which also correlates with decreased virulence *in vivo* that we have previously described (3). The overall altered and generally decreased bacterial dissemination pattern also leads to different disease pathogenesis as observed via histology and IHC visualization of fixed tissues. Histological findings reported here were generally consistent with published data of aerosolized *B. pseudomallei* exposure in mice (37). As described previously, the wild-type ATS2021 strain colonized the brain via the olfactory nerves and olfactory bulb, ultimately resulting in significant destruction of brain tissue (3). In this study, there was an appreciable difference between the pathology associated with the wild-type ATS2021 strain and the mutant *bimA_Bp_* bacteria. The *bimA_Bp_* mutant bacteria still gained access to the brain via the olfactory bulb, but the level of destruction and overall dissemination into the brain tissues were substantially reduced when compared to the wild-type ATS2021 bacteria. For example, the *bimA_Bp_* mutant bacteria were not readily found in the spinal column or vertebral bone marrow despite the higher delivered dose of mutant bacteria, and the damage caused in the brain tissue was markedly less than that observed in mice exposed to aerosols of wild-type ATS2021 bacteria. Taken together, the bacterial dissemination patterns and histology findings support the concept that the presence of the *bimA_Bp_* allele results in a bacterium that causes less severe neurological disease.

Since the different *bimA* alleles promote significant differences in bacterial dissemination and disease pathogenesis, the effects of these alleles were also assessed in the context of the host’s immune response. The prevailing trend with tissue cytokines was for pro-inflammatory cytokines to track bacterial burden, reaching higher levels with wild-type ATS2021 infection compared to the infection initiated by the *bimA_Bp_* mutant. However, this trend was not observed in the mice receiving a very high inoculum of either (10^4^ CFU target dose), where both bacteria and cytokines were higher with the *bimA_Bp_* mutant. Furthermore, cytokine levels appeared to peak earlier in infection with the *bimA_Bp_* mutant. This was likely due to better control of infection and inflammation in mice infected with the mutant bacteria, but may be complicated by selection bias, given that mortality at later time points meant that the mice chosen for analysis were among those that survived longer.

Identifying factors that were differentially expressed early in brain infection could help elucidate differences between the strains in bacterial migration to the central nervous system (CNS). All three challenge doses examined (10^2^, 10^3^, and 10^4^ CFU target dose groups) induced cytokines above baseline on day 1 with wild-type ATS2021 but not the *bimA_Bp_* mutant (i.e., IL-17A, IL-18, IL-23, and IL-28). IL-23 and IL-28 were not detected at all in the brains of mice challenged with the *bimA_Bp_* mutant; IL-17A and IL-18 were detected in mice infected with *bimA_Bp_* mutant bacteria, but with delayed expression compared to wild-type ATS2021.

IL-17A, IL-18, and IL-23 are all pro-inflammatory. IL-28 (IFN-λ) is part of the Type III interferon family, with IFN-α/β-like effects mostly in epithelium, whose role in the CNS is not well-characterized, although it is reported that IL-28 maintains the integrity of the blood-brain barrier by tightening the endothelial junctions in the contexts of viral infections, thereby reducing viral infiltration (38–40). IL-23 and IL-17A are closely related as part of an “axis” in which IL-23 induces CD4 T cell differentiation into IL-17A-secreting Th17 cells. Th17 cells are often helpful in combating bacteria via mechanisms like recruiting neutrophils, releasing extracellular traps, and secreting bactericidal IL-26 (41–43). However, they are implicated in autoimmune diseases in several tissues, including the demyelinating condition multiple sclerosis and its mouse model, experimental autoimmune encephalomyelitis (43–45). IL-23 is produced by astrocytes and microglia, and microglia can also express IL-17 and the IL-23 receptor, potentially reinforcing the inflammatory effect (46, 47).

IL-18 is secreted after inflammasome activation in myeloid immune cells, like IL-1β. However, they are induced by different stimuli: IL-18 is more associated with neurodegenerative disease and with inducing IFN-γ, while IL-1β is associated with generalized inflammation, fever, and cytokine storm (48–50). IL-18 is also expressed constitutively as a precursor and thus can be secreted quickly (e.g., in models of brain injury such as ischemic stroke and intracranial hemorrhage, IL-18 is initially secreted by neurons, then later by glial cells) (51, 52).

Another factor induced above baseline on day 1 was IL-10, an archetypal anti-inflammatory cytokine, which is induced by a wide range of stimuli in microglia, contributing to the control of inflammation in the CNS (53). Unlike other cytokines, IL-10 was at or near peak on day 1 post-exposure in mice exposed to either strain of bacteria (higher with wild-type ATS2021) and remained elevated at later time points.

Two cytokines, IL-27 and IP-10/CXCL10, displayed unique profiles with rapid upregulation in brains harboring the *bimA_Bp_* mutant bacteria, but not wild-type ATS2021 bacteria. Later, 48–72 h post-exposure to wild-type ATS2021 bacteria, the levels of IP-10/CXCL10 surpassed the levels in mice exposed to the *bimA_Bp_* mutant. IL-27 is a member of the IL-12 family, predominantly produced by antigen-presenting cells such as microglia, and like other IL-12 cytokines, its receptor uses JAK/STAT signaling to polarize CD4 T cells toward Th1 while inhibiting Th2 and Th17. The effects of IL-27 on inflammation have been challenging to describe, being strongly pro- or anti-inflammatory depending on the context (54, 55). In the CNS, however, IL-27 is consistently anti-inflammatory and neuroprotective (56). It helps control multiple sclerosis by inhibiting inflammation from Th17, Th9, and DCs (57–59), and suppresses autoimmune uveitis (60). When human astrocytes were exposed to IL-27 under either “inflamed” (additional IL-1β) or “un-inflamed” conditions, *Cxcl10* was among the few genes induced by IL-27 in both conditions (61). Microglia stimulated by IL-27 also produce neuroprotective factors (62). Interestingly, IL-27 has a unique structural similarity to ciliary neurotrophic factor, a secreted factor with important metabolic roles, including maintaining neuronal health and myelination (63, 64).

IFN-γ induces CXCL10, which, in turn, attracts activated T cells and NK cells to sites of inflammation (65). In a comparison of bacterial meningitis to aseptic meningitis patients, both groups had similar CXCL10 and IFN-γ upregulation in cerebrospinal fluid, whereas most other inflammatory mediators were higher when bacteria were present (66). IFN-γ is essential for combating *B. pseudomallei* infection and many other intracellular pathogens (67, 68). IFN-γ was one of the few cytokines where our challenge with ATS2021 wild-type bacteria did not, at any point, lead to higher levels in lungs than challenge with the *bimA_Bp_* mutant, and in fact, expression was higher on day 5 after exposure to the mutant bacteria. CXCL10 is associated with survival in viral infections, notably dengue (69, 70), West Nile, herpesviruses, and coronaviruses (71–75). Although it usually plays a protective role, elevated CXCL10 has been discussed as a biomarker of severity in various viral, bacterial, and parasitic infections (76, 77). Several human pathogens associated with elevated blood levels of CXCL10 are intracellular bacteria, including *B. pseudomallei* itself, as well as *Legionella pneumophila*, *Mycobacterium tuberculosis*, *Mycoplasma pneumoniae*, and *Orientia tsutsugamushi* (78–82), and several intracellular pathogens evade immunity through downregulation of CXCL10 (83).

Chemokines CXCL9, -10, and -11 all signal through the receptor CXCR3, have similar structures, and are often upregulated together by IFN-γ. However, CXCL10 expression can be induced by a wider range of stimuli than CXCL9 and -11, including IL-27, TNF-α, hypoxia, and activators of p38, JNK, ERK1/2, Akt, and NF-κB pathways (61, 84, 85). CXCR3 is expressed on astrocytes, microglia, neurons, and oligodendrocytes, allowing the induction of CXCL10 (65, 86–88). CXCL10 is often induced earlier than most cytokines during viral infection of the CNS and has been called a “sentinel” molecule in this context (65, 86–90). Immune activation by CXCL10 secreted by infected neurons has been observed with varicella zoster virus and rabies virus (86, 91); the latter is a rare case in which blood-brain barrier permeability enhances survival, as it enables a T cell response against rabies virus that would otherwise be hidden from the immune system. CXCL10-induced blood-brain barrier permeability has been seen elsewhere, worsening disease severity (84, 92). It is possible that since some blood-brain barrier disruption is inevitable in neurological melioidosis, early production of factors like IL-27 and CXCL10 could polarize the response toward a relatively less damaging immune infiltrate. Finally, independent of immune signaling, CXCL9, -10, and -11 are notable among chemokines for their bactericidal properties, including against multi-drug-resistant bacteria and spores, and are being studied as sources of antimicrobial peptides for therapy (93). These data suggest that the two strains of bacteria with different *bimA* alleles induce distinct neuroinflammatory states prior to bacterial colonization of the brain, shaping the course of disease progression.

In comparing gene expression trends as identified in NanoString analyses in mice infected with the mutant *bimA_Bp_* strain relative to wild-type ATS2021, we noted some similarities but also several key differences that may explain observed differences in pathogenic phenotype and host immune response. While both isolates elicited broad inflammatory changes in the host (i.e., inflammatory signaling, astrocyte activation, etc.) and suppressed oligodendrocyte function as previously reported for wild-type ATS2021 (3), the relative magnitude of these differences appeared more robust in mice infected with the wild-type ATS2021 strain relative to mice infected with the *bimA_Bp_* mutant. As time passed post-challenge and mice entered the severe disease phase, we saw a general trend of divergence both regarding enriched pathways and individual genes. Several genes that stood out between the two isolates included *Lcn2*, *Ccl2*, and *Gpr34*. In the host, LCN2 has a multifaceted role across multiple organ systems. In its broadest role, it acts as a bacteriostatic acute phase protein due to its ability to sequester iron. In the brain, however, it has a well-known function as a key regulator of neuroinflammation (94–97). Another host gene that stood out between these two isolates is *Ccl2* (encoding monocyte chemoattractant protein 1, or MCP-1); this gene encodes a cytokine that has a broad systemic immune-modulatory role but also is a key player in the brain’s inflammatory response, where it recruits monocytes and macrophages. CCL2 is often closely coexpressed with CCL7, which was overexpressed in brain homogenates infected with wild-type ATS2021 compared to ATS2021 *bimA_Bp_* (98). The apparent higher upregulation of *Ccl2* over time in the wild-type ATS2021 challenge groups relative to the *bimA_Bp_* mutant could suggest a much more prominent role of microglial activation in host brains by bacteria possessing the *bimA_Bm_* phenotype. Monocyte infiltration and activation in diseased or damaged brains is a well-documented phenomenon, and while it can contribute to host disease resolution, an overwhelming host immune response in these organ systems can also have deleterious effects, the classic example being multiple sclerosis (99–103). While there were no appreciable differences in the number of microglia in brain tissues in this study, we did observe evidence that the wild-type ATS2021 bacteria activated a higher percentage of microglia compared to the mutant *bimA_Bp_* bacteria in the brains. Monocyte expansion into the CNS and consequent neuroinflammation in the brain have also been documented in several infectious diseases, including Zika virus (104) and the parasite *Toxoplasma gondii* (105). Finally, and of note, was the more pronounced downregulation of the gene *Gpr34* that we observed in the brains of mice infected with wild-type ATS2021 vs the *bimA_Bp_* mutant bacteria. Gpr34 (G protein-coupled receptor 34) is highly expressed in microglia. Generally, upregulation of this gene correlates with higher levels of neuroinflammation and with potentially poor outcomes, and it has been explored as a target for therapeutics to reduce neuroinflammation (105). Lower Gpr34 expression in the more pro-inflammatory environment observed in mice infected with the wild-type ATS2021 would not be unprecedented, however, as it has been reported that both dendritic cells and microglia downregulate the expression of Gpr34 in the presence of LPS (and other stimuli) *in vitro*, and this lower expression has been associated with increased apoptosis in dendritic cells, which also reside in the brain (106, 107).

Overall, the host gene expression data presented here comparing wild-type ATS2021 and *bimA_Bp_* mutant bacteria strongly suggest a scenario wherein the wild-type ATS2021 isolate has a unique ability, perhaps owing to a neuroinvasive phenotype, to stimulate rapidly amplifying inflammatory responses in the murine brain. These ultimately fatal host responses are distinguished by the activation of numerous pro-inflammatory markers, including LCN2 and CCL2, the latter of which might also signify a role for monocyte infiltration into the CNS as part of this deleterious immune response. It is worth noting that LCN2 and CCL2 are both under investigation as potential therapeutic targets across a range of neuroinflammatory diseases (94, 108–110), and that these or other therapies to dampen inflammation could be investigated in the future as potential approaches for combating the harmful neurological effects of *B. pseudomallei* infection.

As previously described, the effects of neurological melioidosis can be devastating (29, 111–113). This neurological aspect of disease is important in the context of both public health and biodefense scenarios but remains poorly understood. By elucidating the pathogenesis of the bacterium and the mechanisms used by the host to combat infection, particularly once the bacteria are in the brain, we could discover or potentially repurpose existing medical countermeasures to specifically target neurological melioidosis. These immune and transcriptomic profile data could also be used to potentially refine novel diagnostic strategies in the future to combat new or reemerging microbial pathogens that result in neurological sequelae.

## SUPPLEMENTAL MATERIAL

Supplemental material iai.00727-25-s0001.pdf Fig. S1 to S10; Table S4.

Supplemental tables iai.00727-25-s0002.xlsx Tables S1 and S2.

Table S3 iai.00727-25-s0003.xlsx NanoString panel gene list.

Table S5 iai.00727-25-s0004.xlsx Wild-type ATS2021 attributes and normalized counts.

Table S6 iai.00727-25-s0005.xlsx *bimA_Bp_* mutant ATS2021 attributes and normalized counts.

Table S7 iai.00727-25-s0006.xlsx Wild-type ATS2021 DEGs and pathway scores.

Table S8 iai.00727-25-s0007.xlsx *bimA_Bp_* mutant ATS2021 DEGs and pathway scores.

ASM does not own the copyrights to Supplemental Material that may be linked to, or accessed through, an article. The authors have granted ASM a non-exclusive, world-wide license to publish the Supplemental Material files. Please contact the corresponding author directly for reuse.
