# Supplementary material for: Characterization of an isogenic bimA mutant in the ATS2021 strain of Burkholderia pseudomallei
Source: Infect Immun. 2026 Jun 18;94(7):e00727-25. doi: 10.1128/iai.00727-25 (PMC13367048; doi:10.1128/iai.00727-25)
Supplement: Supplemental material — Fig. S1 to S10; Table S4. [file iai.00727-25-s0001.pdf]

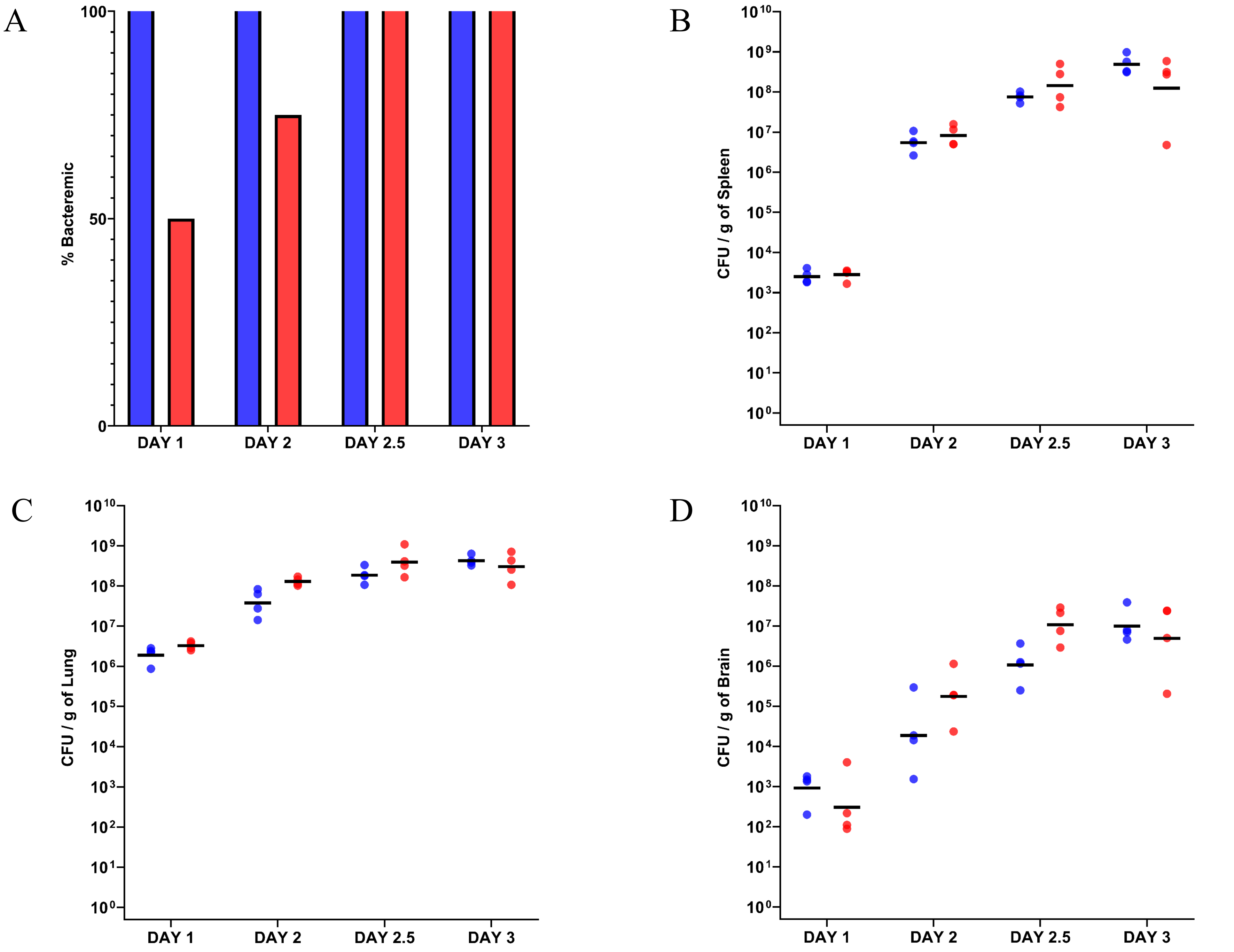

Figure S1. Evaluation of bacterial burden in select tissues three days post-exposure to a high-dose aerosolized bacteria. Mice were exposed to the 10^4 CFU target dose, 15,566 CFU of aerosolized ATS 2021 *bimA*$_{Bp}$ (red) and then mice were serially sampled on days 1, 2, 2.5, and 3 to determine % of mice that were bacteremic at that time point (**A**), bacterial burdens in spleens (**B**), lungs (**C**), and brains (**D**). These bacterial burdens were compared to previously published data detailing the bacterial burden after exposure to 4,488 CFU of aerosolized CFU of the wild-type ATS2021 strain (blue). For CFU data, pairwise treatment groups were compared by linear mixed effects model. No multiplicity adjustment was applied.

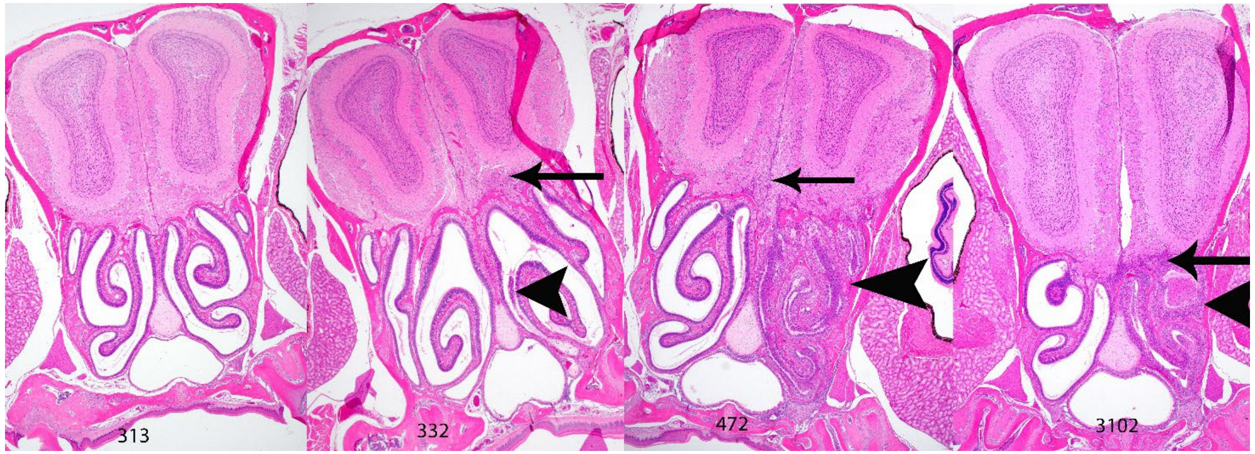

Figure S2. Representative histopathological analyses from mice challenged with approximately 204 aerosolized CFU of ATS2021 $bimA_{Bp}$. Head sections with nasal cavity including nasal turbinates, nasal septum, respiratory and olfactory epithelium, lamina propria with supporting tissues and glands, nerve bundles, and nasal air passages; cribriform plate (bone and olfactory/trigeminal nerves); and cranial vault with olfactory bulb histopathology from days 1 (313), 3 (332), 7 (372), and 10 (3102) PE. **313**-Tissues are normal. HE 2X **332**-There is mild necrosuppurative rhinitis (arrowhead) and minimal neutrophilic inflammation of the olfactory nerves (arrow). HE 2X **472**- There is moderate necrosuppurative rhinitis (arrowhead) and minimal neutrophilic inflammation of the olfactory nerves (arrow). HE 2X **3102**- There is moderate necrosuppurative rhinitis (arrowhead) and minimal and mild neutrophilic inflammation of the olfactory nerves and olfactory bulb (arrow). HE 2X

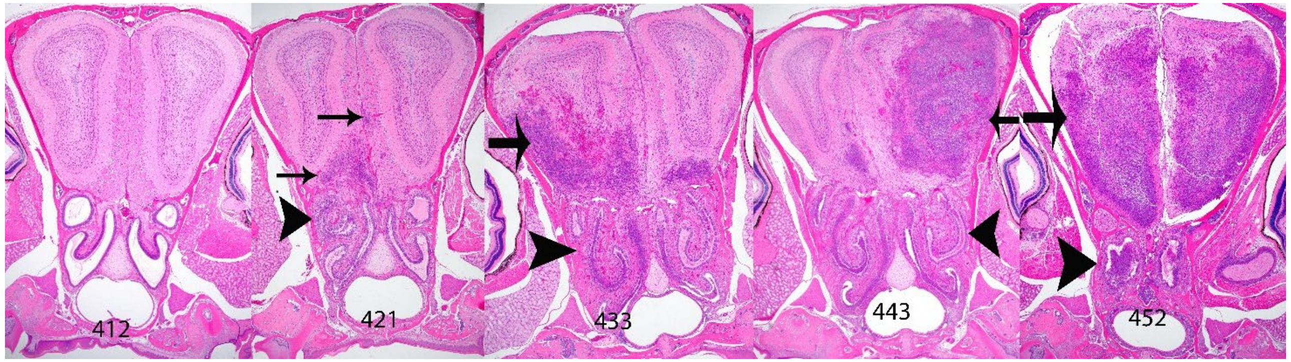

Figure S3. Representative histopathological analyses from mice challenged with approximately 1,563 aerosolized CFU of ATS2021 $bimA_{Bp}$. Head sections with nasal cavity including nasal turbinates, nasal septum, respiratory and olfactory epithelium, lamina propria with supporting tissues and glands, nerve bundles, and nasal air passages; cribriform plate (bone and olfactory/trigeminal nerves); and cranial vault with olfactory bulb histopathology from days 1 (412), 2 (421), 3 (433), 4 (443), and 5 (452) PE. **412**-Tissues are normal. HE 2X **421**-There is moderate necrosuppurative rhinitis (arrowhead) and moderate and necrotizing and suppurative inflammation of the olfactory nerves and olfactory bulb (arrow). HE 2X **433**- There is marked necrosuppurative rhinitis (arrowhead), marked and necrotizing and suppurative inflammation of the olfactory nerves, and moderate hemorrhagic meningoencephalitis of the olfactory bulb (arrow). HE 2X **443**- There is marked necrosuppurative rhinitis (arrowhead), marked and necrotizing and suppurative inflammation of the olfactory nerves, and marked hemorrhagic meningoencephalitis of the olfactory bulb (arrow). HE 2X **452-**There is severe necrosuppurative rhinitis (arrowhead), severe and necrotizing and suppurative inflammation of the olfactory nerves, and severe hemorrhagic meningoencephalitis of the olfactory bulb (arrow). HE 2X

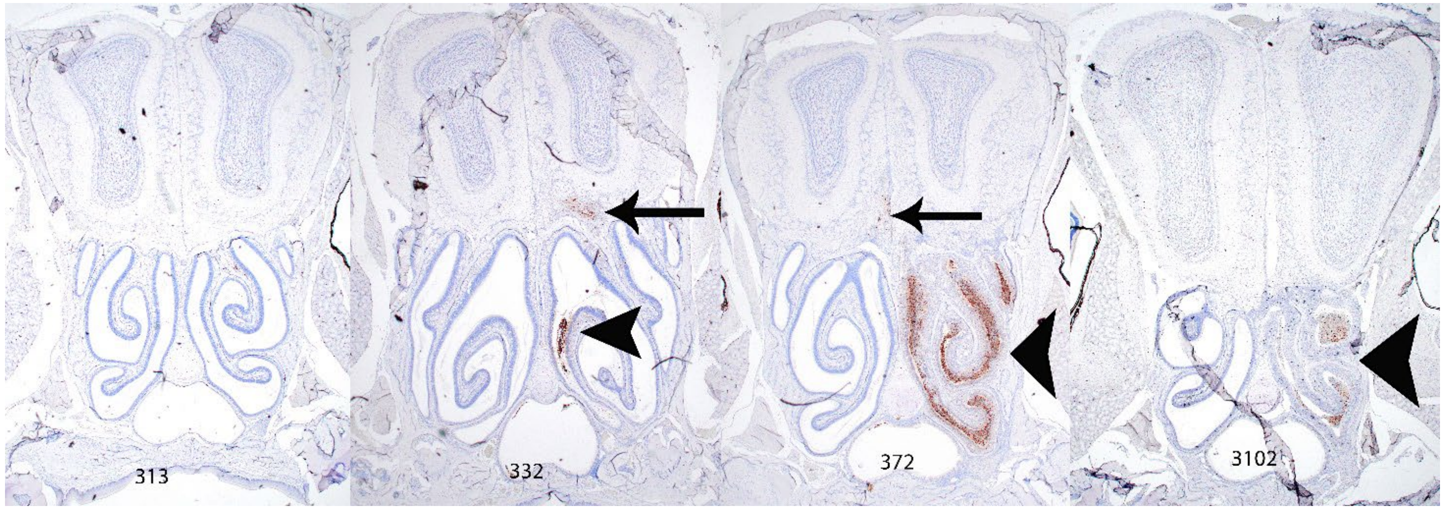

Figure S4. Representative immunohistochemical analyses from mice challenged with approximately 204 aerosolized CFU of ATS2021 $bimA_{Bp}$. Head sections with nasal cavity including nasal turbinates, nasal septum, respiratory and olfactory epithelium, lamina propria with supporting tissues and glands, nerve bundles, and nasal air passages; cribriform plate (bone and olfactory/trigeminal nerves); and cranial vault with olfactory bulb immunohistochemistry from days 1 (313), 3 (332), 7 (372), and 10 (3102) PE. **313**-negative **332**, **372**, and **3102** display mild to moderate IHC positivity of nasal turbinates (arrowheads) and olfactory bulb (arrows). *Burkholderia* IHC 2X

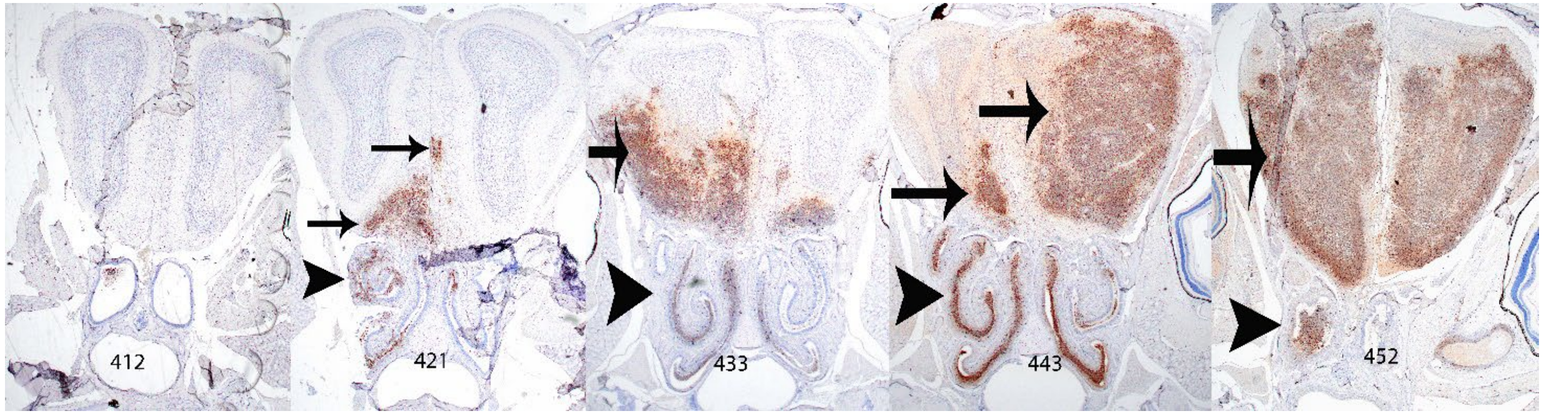

Figure S5. Representative immunohistochemical analyses from mice challenged with approximately 1,563 aerosolized CFU of ATS2021 $bimA_{Bp}$. Head sections with nasal cavity including nasal turbinates, nasal septum, respiratory and olfactory epithelium, lamina propria with supporting tissues and glands, nerve bundles, and nasal air passages; cribriform plate (bone and olfactory/trigeminal nerves); and cranial vault with olfactory bulb immunohistochemistry from days 1 (412), 2 (421), 3 (433), 4 (443), and 5 (452) PE. **412**-negative, **421**, **433**, **443**, and **452** display moderate to severe IHC positivity of nasal turbinates (arrowheads) and olfactory nerves and olfactory bulb (arrows). *Burkholderia* IHC 2X

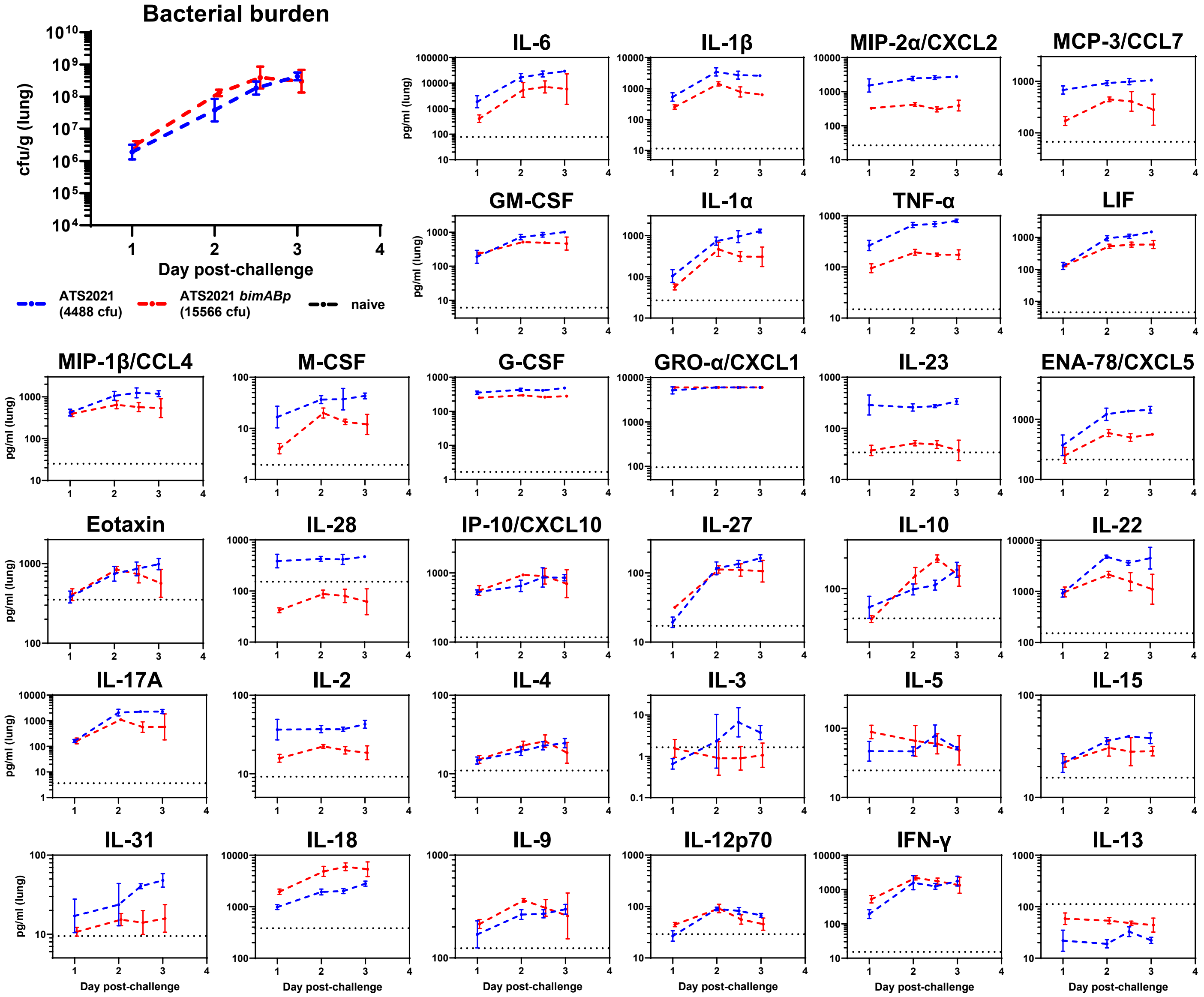

Figure S6. Evaluation of cytokine response in lung homogenates three days post-exposure to a mid-dose or aerosolized *B. pseudomallei*. Cytokine levels in lung homogenates from mice exposed to the 10^4 CFU target dose, 4,488 wild-type ATS2021 CFU (blue) or 15,566 CFU mutant ATS2021 $bimA_{Bp}$ CFU (red) at days 1, 2, 2.5, and 3 post-challenge (n = 4). Cytokines are sorted from the greatest difference (wild-type ATS2021 compared to mutant ATS2021 $bimA_{Bp}$) at day 5 post-challenge. Data is shown as geometric means with error bars representing the geometric standard deviation. Black dotted line indicates the geomean of cytokine levels in naïve lung homogenates (n = 4). For Luminex data, pairwise treatment groups were compared by linear mixed effects model. No multiplicity adjustment was applied.

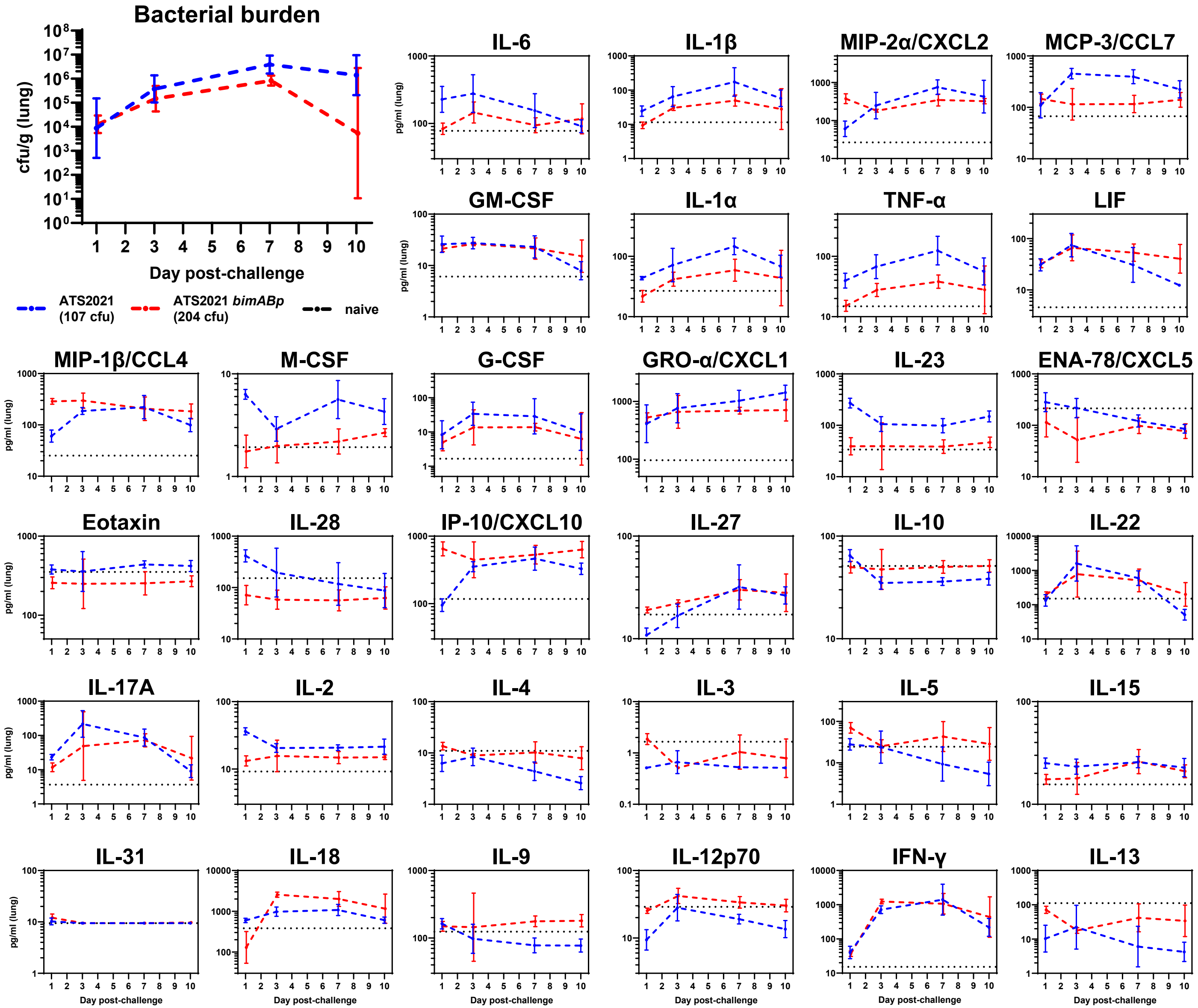

Figure S7. Evaluation of cytokine response in lung homogenates three days post-exposure to a mid-dose or aerosolized *B. pseudomallei*. Cytokine levels in lung homogenates from mice exposed to the 10^2 CFU target dose, 104 CFU of wild-type ATS2021 (blue) or 204 CFU of mutant ATS2021 $bimA_{Bp}$ (red) at days 1, 3, 7, and 10 post-challenge (n = 4). Cytokines are sorted from the greatest difference (wild-type ATS2021 compared to mutant ATS2021 $bimA_{Bp}$) at day 5 post-challenge. Data is shown as geometric means with error bars representing the geometric standard deviation. Black dotted line indicates the geomean of cytokine levels in naïve lung homogenates (n = 4). For Luminex data, pairwise treatment groups were compared by linear mixed effects model. No multiplicity adjustment was applied.

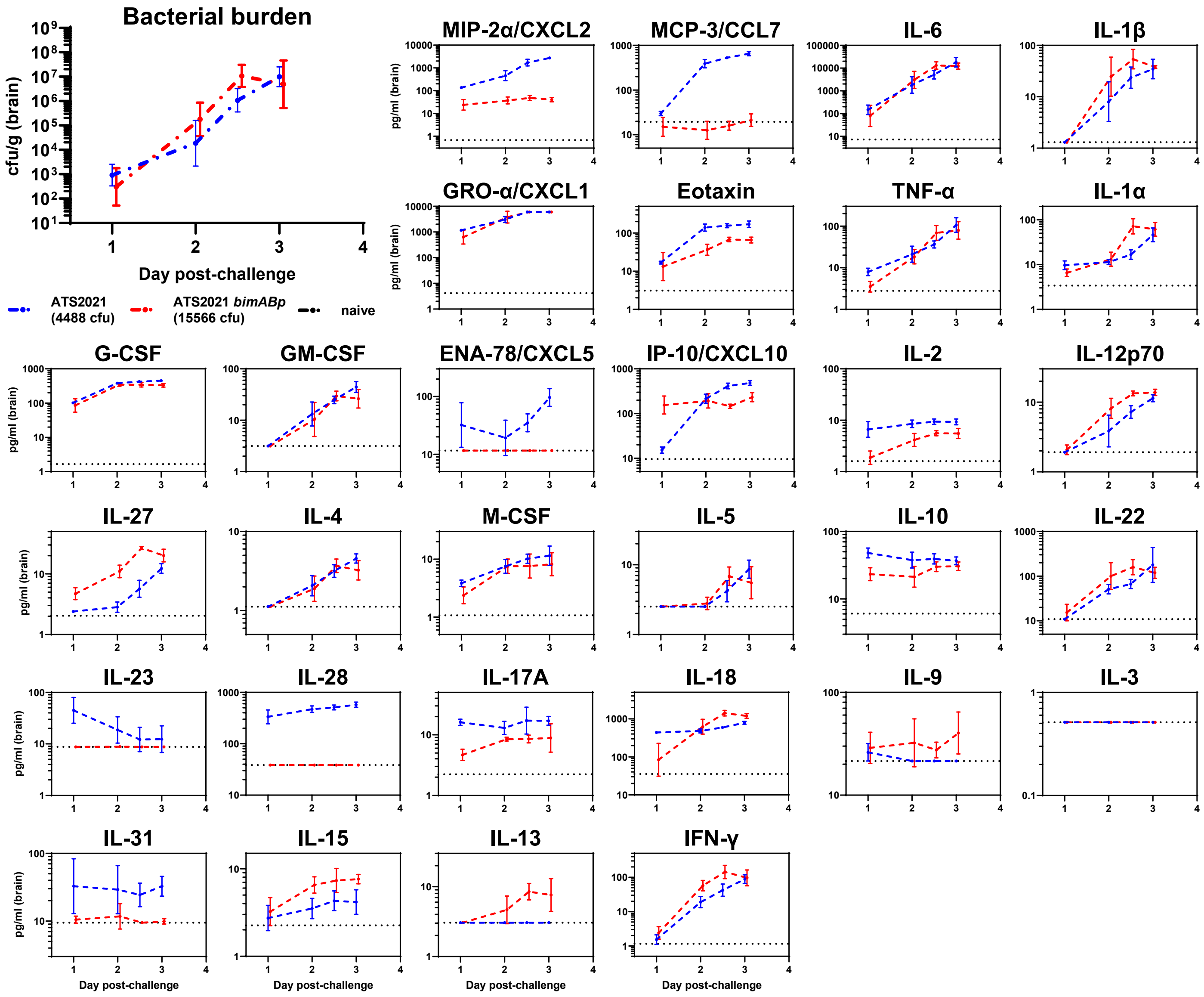

Figure S8. Evaluation of cytokine response in brain homogenates three days post-exposure to a mid-dose or aerosolized *B. pseudomallei*. Cytokine levels in brain homogenates from mice exposed to the 10^4 CFU target dose, 4,488 CFU of wild-type ATS2021 (blue) or 15,566 CFU of mutant ATS2021 $bimA_{Bp}$ (red) at days 1, 2, 2.5, and 3 post-challenge ($n = 4$). Cytokines are sorted from the greatest difference (wild-type ATS2021 compared to mutant ATS2021 $bimA_{Bp}$) at day 5 post-challenge. Data is shown as geometric means with error bars representing the geometric standard deviation. Black dotted line indicates the geomean of cytokine levels in naïve lung homogenates ($n = 4$). For Luminex data, pairwise treatment groups were compared by linear mixed effects model. No multiplicity adjustment was applied.

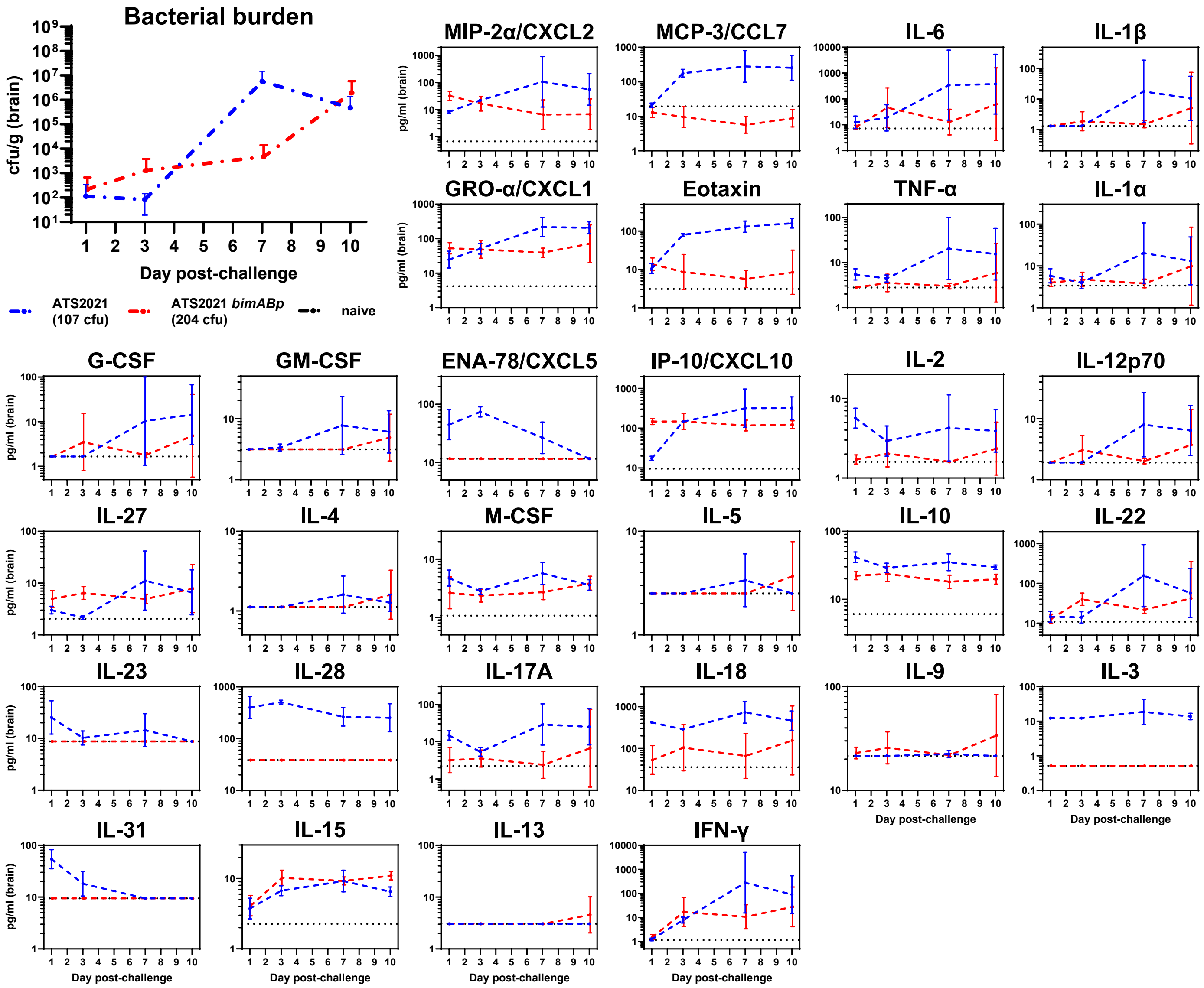

Figure S9. Evaluation of cytokine response in brain homogenates three days post-exposure to a mid-dose or aerosolized *B. pseudomallei*. Cytokine levels in brain homogenates from mice exposed to the 10^2 CFU target dose, 107 CFU of wild-type ATS2021 (blue) or 204 CFU of mutant ATS2021 $bimA_{Bp}$ (red) at days 1, 3, 7, and 10 post-challenge ($n = 4$). Cytokines are sorted from the greatest difference (ATS2021 compared to ATS2021 $bimA_{Bp}$) at day 5 post-challenge. Data is shown as geometric means with error bars representing the geometric standard deviation. Black dotted line indicates the geomean of cytokine levels in naïve lung homogenates ($n = 4$). For Luminex data, pairwise treatment groups were compared by linear mixed effects model. No multiplicity adjustment was applied.

Table S4. Summary of significantly differentially expressed genes in brains of C57BL/6 mice challenged with various target doses of either aerosolized wildtype *bimA*$_{Bm}$ or mutant *bimA*$_{Bp.}$ Significant genes are divided between up and downregulated groups.

Day Post-Challenge	***bimA**$_{Bm}$* **- 107 CFU**		***bimA**$_{Bp}$* **- 204 CFU**		***bimA**$_{Bm}$* **- 1,563 CFU**		***bimA**$_{Bp}$* **- 1,149 CFU**		***bimA**$_{Bm}$* **- 4,488 CFU**		***bimA**$_{Bp}$* **- 15,566 CFU**	
	Up	Down	Up	Down	Up	Down	Up	Down	Up	Down	Up	Down
1	8	17	1	25	28	13	15	22	41	50	22	63
2	NC[1]	NC	NC	NC	79	43	84	46	82	108	115	69
3	21	5	30	5	135	32	68	9	115	101	130	73
4	NC	NC	NC	NC	184	77	202	35	110	115	X	X
5	NC	NC	NC	NC	207	170	45	25	X[2]	X	X	X

Significant genes are divided between up and downregulated groups.

[1]NC denotes that samples were not collected on that day

[2]X denotes animals that succumbed to infection or were euthanized in accordance with early endpoint euthanasia criteria

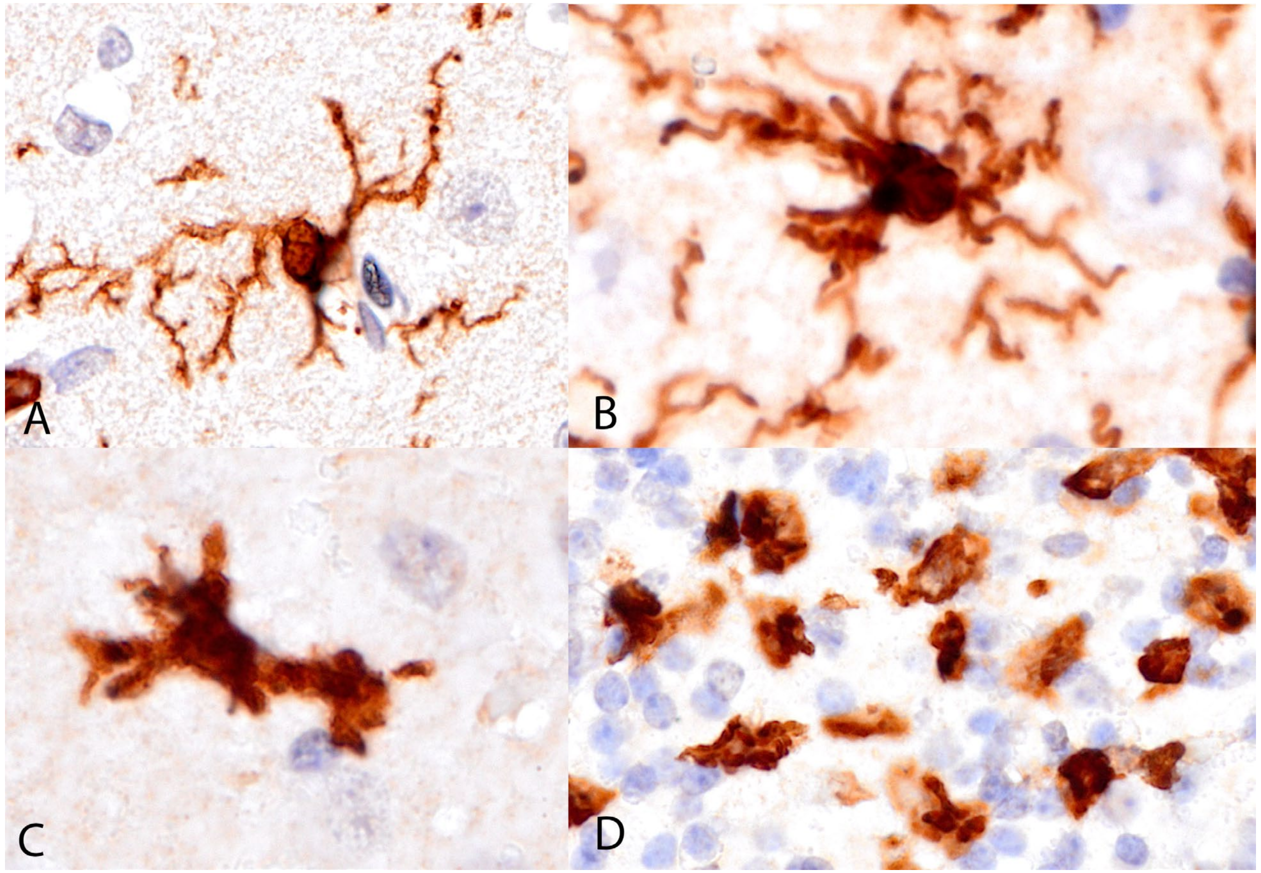

Figure S10. Representative immunohistochemical analyses from mice challenged with aerosolized *B. pseudomallei*. Examples of microglial activation as determined by IHC staining. Mouse 4 Cerebrum infected with *B. pseudomallei* ATS2021 wild-type. Ramified microglia with cell body and cell processes. Iba1 60X (A); Mouse 432 Cerebrum infected with *B. pseudomallei* ATS2021 $bimA_{Bp}$. Hyper-ramified microglia with increased branching of cell processes. Iba1 60X (B); Mouse 39 Cerebrum infected with *B. pseudomallei* ATS2021 wild-type. Bushy microglia with retracted and thickened cell processes. Iba1 60X (C); and Mouse 432 Olfactory bulb infected with *B. pseudomallei* ATS2021 $bimA_{Bp}$. Amoeboid microglia are fully activated and are capable of phagocytosis. Iba1 60X (D)
